# Contrasted patterns of local adaptation to climate change across the range of an evergreen oak, *Quercus aquifolioides*

**DOI:** 10.1111/eva.13030

**Published:** 2020-06-09

**Authors:** Fang K. Du, Tianrui Wang, Yuyao Wang, Saneyoshi Ueno, Guillaume de Lafontaine

**Affiliations:** ^1^ School of Ecology and Nature Conservation Beijing Forestry University Beijing China; ^2^ Department of Forest Molecular Genetics and Biotechnology Forestry and Forest Products Research Institute Forest Research and Management Organization Tsukuba Japan; ^3^ Canada Research Chair in Integrative Biology of Northern Flora Université du Québec à Rimouski Rimouski QC Canada

**Keywords:** adaptation, Fagaceae, genomic divergence, Hengduan Mountains, landscape genomics, *Quercus*, Tibet

## Abstract

Long‐lived tree species are genetically differentiated and locally adapted with respect to fitness‐related traits, but the genetic basis of local adaptation remains largely unresolved. Recent advances in population genetics and landscape genomic analyses enable identification of putative adaptive loci and specific selective pressures acting on local adaptation. Here, we sampled 60 evergreen oak (*Quercus aquifolioides*) populations throughout the species' range and pool‐sequenced 587 individuals at drought‐stress candidate genes. We analyzed patterns of genetic diversity and differentiation for 381 single nucleotide polymorphisms (SNPs) from 65 candidate genes and eight microsatellites. Outlier loci were identified by genetic differentiation analysis and genome–environment associations. The response pattern of genetic variation to environmental gradient was assessed by linear isolation‐by‐distance/environment tests, redundancy analysis, and nonlinear methods. SNPs and microsatellites revealed two genetic lineages: Tibet and Hengduan Mountains–Western Sichuan Plateau (HDM‐WSP), with reduced genetic diversity in Tibet lineage. More outlier loci were detected in HDM‐WSP lineage than Tibet lineage. Among these, three SNPs in two genes responded to dry season precipitation in the HDM‐WSP lineage but not in Tibet. By contrast, genetic variation in the Tibet lineage was related to geographic distance instead of the environment. Furthermore, risk of nonadaptedness (RONA) analyses suggested HDM‐WSP lineage will have a better capacity to adapt in the predicted future climate compared with the Tibet lineage. We detected genetic imprints consistent with natural selection and molecular adaptation to drought on the Qinghai–Tibet Plateau (QTP) over a range of long‐lived and widely distributed oak species in a changing environment. Our results suggest that different within‐species adaptation processes occur in species occurring in heterogeneous environments.

## 1 INTRODUCTION

Climatic oscillations can have a profound impact on biodiversity and sustainability at every level of the biota, from genes to ecosystems (de Lafontaine, Napier, Petit, & Hu, 2018; Hewitt, 2000; IPCC, 2013). In response to rapid climate change, natural populations can migrate to new favorable locations, adapt locally to novel environments, or become extinct (Aitken, Yeaman, Holliday, Wang, & Curtis‐McLane, 2008; Savolainen, Pyhäjärvi, & Knürr, 2007; Sork et al., 2010). Among these responses, local adaptation to altered environments seems especially crucial for sedentary and long‐lived organisms such as forest tree species, because moving to favorable locations through propagule dispersal might not be fast enough to cope with the rate of ongoing climate change (Corlett & Westcott, 2013; Hughes, Inouye, Johnson, Underwood, & Vellend, 2008; Kremer et al., 2012; Sork et al., 2013).

Long‐lived tree species with large population size are typically locally adapted to different conditions within highly heterogeneous environments (Savolainen, 2011). Recent advances in ecological genomics of nonmodel species have started to unravel the molecular genetic basis for local adaptation in tree species (Sork et al., 2013). Numerous methods are available, and all of them ultimately rely on the rejection of the neutral model of evolution to detect genomic imprints of natural selection (Hoban et al., 2016). One approach infers natural selection by comparing the strength of population structure among loci (*F*_ST_ outlier analysis) (Lotterhos & Whitlock, 2014). Another approach estimates the strength of associations between environmental variables in structuring genetic variation by relying on genotype–environment associations (GEAs), tests of isolation‐by‐environment, constrained ordination techniques, or generalized dissimilarity modeling (Sork, 2018). Jointly using these various methods to study the molecular imprint of local adaptation is likely to provide the best inferences because these approaches are conceptually distinct, with different set of assumptions and computational frameworks.

The Qinghai–Tibet Plateau (QTP) in southwest China is the largest and highest plateau (average elevation > c. 4,000 m) in the world (Zhang, Ye, & Sun, 2016), with the Himalaya–Hengduan Mountains considered as one of the World's biodiversity hotspots (Marchese, 2015; Myers, Mittermeier, Mittermeier, Da Fonseca, & Kent, 2000). In recent years, numerous phylogeographical surveys have examined more than 80 plant species in the QTP and its surroundings (reviewed by Yu et al., 2018). These studies focused on neutral genetic variation to investigate population dynamics and biogeographic history, while overlooking plant adaptive potential in relation to the local environment. Yet, organisms within natural populations inhabiting the QTP are likely to be locally adapted to the high altitude, an extreme environment of the plateau. Rapid desertification, directly caused by an increasingly drier and warmer climate (Xue, Guo, Han, Sun, & Liu, 2009) in the QTP, should trigger detectable adaptive responses in plants. Specifically, drought (water deficit) represents a critical condition, which imposes a strong selective pressure fostering rapid local adaptation in plants (e.g., Eveno et al., 2008; Petit et al., 1999). Investigating drought adaptive response of long‐lived trees is timely because most climate‐change scenarios suggest a general increase in aridity worldwide (e.g., Park, Sur, Kim, & Lee, 2018).

*Quercus* L. (oak) is one of the most diverse and ecologically important tree genera in the Northern Hemisphere, with high species diversity in Central America and South‐East Asia (Denk, Grimm, Manos, Deng, & Hipp, 2018). Because of its tolerance to a wide range of environments and contrasting habitats (Xu, Dimitrov, Shrestha, Rahbek, & Wang, 2019), the oak genus has provided useful insights on evolutionary mechanisms underlying local adaptation (Kremer, 2016; Petit et al., 2013). Landscape genomic studies have revealed patterns of local adaptation in European white oaks (*Quercus petraea*, *Q. pubescens*, *Q. robur*; Rellstab et al., 2016) and North America valley oak (*Q. lobata*; Sork et al., 2010, 2016). The recent release of a high‐quality assembled genomic sequence of *Q. robur* (Plomion et al., 2018) may provide new tools to study genomic imprints of adaptation in oaks. According to the State Forestry Administration of China, oaks comprise 13% of the natural forest in China. Yet, genetic studies on *Quercus* spp. in Asia are limited relative to Europe and North America, and have mainly focused on uncovering neutral genetic structure and phylogeographic patterns (Lyu et al., 2018). Studies specifically addressing local adaptation of oaks in Asia are thus urgently needed.

The Himalaya–Hengduan Mountains biodiversity hotspot of the QTP hosts no less than five evergreen highland oak species (Meng et al., 2017). Among these, the endemic *Quercus aquifolioides* is the most widely distributed and occupies the highest elevation, reaching the tree line in some areas (Du, Hou, Wang, Mao, & Hampe, 2017). According to an updated classification of oaks, *Q. aquifolioides* belongs to subgenus *Cerris* in section *Ilex* (Denk et al., 2018). The species displays exceptional environmental adaptations, including broad tolerance from subtropical humid to cold dry environments in steep high solar radiation slopes of rugged mountains, at an elevation ranging from 1,900 to 4,600 m a.s.l. (Huang, Zhang, & Bartholomew, 1999; Tang, 2006). Because of its wide geographical distribution and ecological amplitude, *Q. aquifolioides* might provide useful insights on the genetic mechanism of adaptive variation. Here, we relied on a joint analysis from state‐of‐the‐art approaches to identify genetic imprints consistent with natural selection and molecular adaptation across the range of *Q. aquifolioides*. To this end, we targeted candidate gene sequences known to be involved in adaptation to drought stress in other oak species from the EvolTree database (http://www.evoltree.eu/index.php/candidate‐genes‐db). We hypothesized that the combination of *F*_ST_ outlier detection methods, SNP annotations, and various procedures testing associations between genetic and environmental variables could identify molecular signatures consistent with local adaptation to drought, a major climatic driver limiting plant growth in the Himalaya–Hengduan Mountains biodiversity hotspot. Furthermore, in order to determine how adaptation would contribute to *Q. aquifolioides* response to ongoing climate change, we predicted the “adaptedness” (i.e., adaptive capacity; Foden et al., 2013) of *Q. aquifolioides* to future local climate based on climate data modeled under a scenario of future climate warming. Our study lays the groundwork for further investigations unraveling ecological adaptation of oaks in Asia and provides new insights into local adaptive response to climatic selective pressure in tree species.

## 2 MATERIALS AND METHODS

### 2.1 Field sampling, DNA isolation, and microsatellite genotyping

**FIGURE 1:**
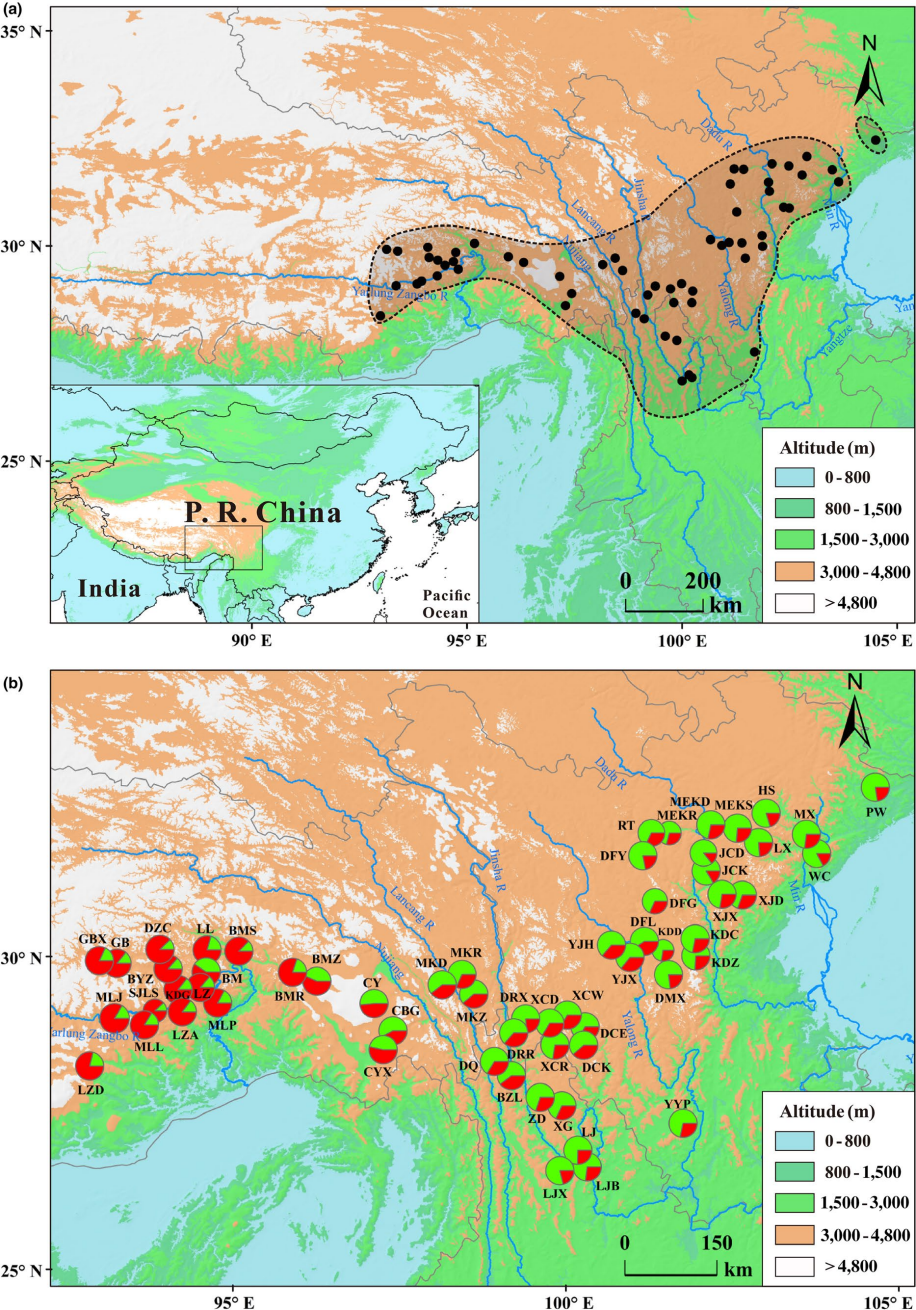
Geographical distribution, location of study sites, and mapping of the Bayesian genetic clusters in *Quercus aquifolioides*. (a) Species range (gray shadow) and sampling locations (black dots). (b) Two main clusters indicated by red (Tibet lineage) and green (HDM‐WSP lineage) based on Bayesian cluster analysis of eight nuclear microsatellite markers from 587 individuals in 60 populations

We sampled leaf material from 60 study sites spaced >30 km apart and covering the range of *Q. aquifolioides* in southwest China as described in the Chinese Virtual Herbarium. At each site, foliage of 7–11 individuals spaced >100 m apart was sampled and stored in silica gel, reaching a total of 587 individuals (Figure 1; Table S1). Total genomic DNA was isolated from dry leaf tissue using a Plant Genomic DNA Extraction Kit (Tiangen). All 587 individuals were genotyped at eight neutral nuclear microsatellite (SSR) markers described by Du et al. (2017). This dataset served as a control, indicative of neutral genetic variation in our sample.

### 2.2 Climatic variables

For each study site, a total of 103 climate variables for current conditions (~1970–2000) and future predictions (2050 and 2070) were extracted from WorldClim Version2 raster layers at 30 s (~1 km^2^) of resolution (Fick & Hijmans, 2017; http://www.worldclim.org/version2). These included the full suite of 19 mean annual bioclimatic variables along with average monthly climate data for precipitation, solar radiation, wind speed, water vapor pressure, and minimum, mean, and maximum temperature. To avoid biased estimates of model coefficients and spurious significance levels resulting from multicollinearity, we excluded highly correlated climate variables with the threshold values of 0.7 using a variance inflation factor (VIF) test in “usdm” R package (Naimi, Hamm, Groen, Skidmore, & Toxopeus, 2014; R Core Team, 2018). Along with the two geographic variables (latitude and longitude), four climatic variables—isothermality (bio03, a measure of monthly temperature oscillation relative to annual temperature oscillation), mean temperature of the driest quarter (bio09), precipitation during the dry season (prec01), and precipitation during the wet season (prec06)—were finally retained for subsequent analyses (Table S1).

### 2.3 Candidate gene fragment selection and annotation

A set of 180 drought‐related stress candidate genes in *Quercus petraea* and *Quercus robur* were downloaded from the EvolTree database (Table S2; http://www.evoltree.eu/index.php/candidate‐genes‐db). We used Primer3web (Untergasser et al., 2012; http://primer3.ut.ee) to design PCR primer pairs for each candidate gene. We tested each pair of primers on eight individuals under various PCR conditions. The PCR products were visually checked on agarose gel and validated by the Sanger sequencing (ABI X3730XL DNA analyzer). We retained 65 polymorphic gene fragments for further genotyping. Sequences were submitted to GenBank and used as a reference in further analyses.

We designed pair‐end barcode sequences to distinguish each individual after pool sequencing (Figure S1). Specifically, 20‐bp barcode sequences (10 bp was connected to the 5′ end of forward and reverse primers, respectively) with a 3‐bp difference were designed to distinguish among 100 individuals through pairwise coupling. We amplified the 65 candidate gene fragments and pooled PCR products in equimolar ratios into six libraries, each including PCR products from ca. 100 individuals. Libraries were sequenced using an Illumina MiSeq System with a paired‐end sequence of 250‐nt reads, at the Center of Biomedical Analysis (Tsinghua University).

Illumina raw reads were split according to barcode information. Low‐quality sequences and Illumina‐specific adapters were removed by Trimmomatic 0.36 (Bolger, Lohse, & Usadel, 2014), and the clean reads were mapped onto the original reference sequences by DNA mapping algorithm Burrow–Wheeler Aligner, BWA‐MEM (Li & Durbin, 2009). Duplicates were marked in each aligned file by Picard Tools (http://broadinstitute.github.io/picard), and the reads around any insertion/deletion (indel) were realigned by the Genome Analysis Toolkit (GATK; Mckenna et al., 2010). The initial SNPs were called, filtered, a new round of SNP calling was performed based on the initial SNPs, and the desired phased haplotypes were produced from our variants. Using VCFtools (Danecek et al., 2011), we retained high‐quality (quality score >30) biallelic SNPs with a minor allele frequency (MAF) ≥2.5% (Rellstab et al., 2016).

We downloaded amino acid sequences for European white oak, *Q. robur* (Plomion et al., 2018) from the OAK GENOME SEQUENCING website (http://www.oakgenome.fr/), and performed a blastx search of the candidate genomic sequences in *Q. aquifolioides* against *Q. robur* amino acid sequences. If the high scoring segment pairs (HSPs) of the blast top hit contained no stop codons, we assumed that the segments were likely to be coding sequences (CDSs). We used SnpEff (Cingolani et al., 2012) to annotate likely gene functions of the variants.

### 2.4 Genetic structure and diversity

Using “adegenet” R package (Jombart & Ahmed, 2011), we conducted a principal component analysis (PCA) to produce a lower‐dimensional subspace that captured most of the variation in each of three datasets: all SNPs, non‐*F*_ST_ outlier SNP subset (i.e., SNPs not detected as *F*_ST_ outliers; see section *F*_ST_ outlier detection below), and SSRs. Based on results of the PCA, we assigned the genetic variation of *Q. aquifolioides* to two known geographic clusters (Du et al., 2017): the Tibet lineage (17 populations) and the HDM‐WSP lineage (43 populations). We estimated the degree of genetic divergence between lineages, among populations within lineages, and within populations with an analysis of molecular variance (AMOVA) in Arlequin 3.5 (Excoffier & Lischer, 2010).

We estimated mean frequency of the most frequent allele at each locus (*P*), mean observed heterozygosity (*H*_O_), mean expected heterozygosity (*H*_E_), and mean nucleotide diversity (*π*) for each lineage based on all SNPs and non‐*F*_ST_ outlier SNPs using STACKS 1.47 (Catchen, Hohenlohe, Bassham, Amores, & Cresko, 2013). For SSRs, the mean effective population size (*N*_E_), mean observed heterozygosity, mean expected heterozygosity, and mean Shannon index (*I*) for each lineage were calculated by GenAlEx 6.5 (Peakall & Smouse, 2012). For each summary statistic, 2‐group Mann–Whitney *U* test was used to evaluate the significance of the difference between the two lineages.

### 2.5 Molecular signature of local adaptation

Various approaches are used to reveal molecular imprints consistent with adaptive evolution. *F*_ST_ outlier analyses scan the genome in search of locus‐specific effects, assumed to reflect diversifying or balanced selection, as revealed by higher (positive outliers) or lower (negative outliers) genetic differentiation (*F*_ST_) compared with the neutral background level, respectively (Beaumont & Balding, 2004; Beaumont & Nichols, 1996; Excoffier, Hofer, & Foll, 2009; Hohenlohe, Phillips, & Cresko, 2010). This approach typically requires large samples from distinct populations (Eveno et al., 2008) and does not account for environmental heterogeneity. To this end, genotype–environment associations (GEAs) flag loci whose allele frequency is strongly correlated with environmental gradients (Coop, Witonsky, Di Rienzo, & Pritchard, 2010; de Villemereuil, Frichot, Bazin, François, & Gaggiotti, 2014; Frichot, Schoville, Bouchard, & François, 2013), often revealing adaptive patterns that are not detected by *F*_ST_ outlier analysis (Rellstab, Gugerli, Eckert, Hancock, & Holderegger, 2015). Contrary to *F*_ST_ outlier and GEAs, multivariate approaches do not search for molecular imprints of locus‐specific adaptive effects. Instead, they can provide insights on the role of adaptation by testing the multivariate relationships between environmental gradients and genetic structure across populations, while accounting for spatial genetic structure caused by neutral evolutionary processes.

#### 2.5.1 *F*_ST_ outlier detection

We combined two methods to identify outlier loci with extremely high *F*_ST_ values, assumed to reflect a locus‐specific imprint of diversifying selection. First, BayeScan relies on a Bayesian approach that directly estimates the posterior probability that a given locus is under selection, taking the effective population size and migration rate into account, thus reducing false positives (Foll & Gaggiotti, 2008). We computed 20 pilot runs with a run length of 5 × 10^3^ and a burn‐in of 5 × 10^4^ followed by 5 × 10^3^ iterations with a thinning interval of 10. Prior odds were set to 2 as suggested for candidate genes (Csilléry et al., 2014; Roschanski et al., 2016). SNPs with posterior odds (PO) >2 were considered as outliers.

Second, FDIST2 implemented in Arlequin 3.5 was used to establish, based on 10^5^ simulations, a null distribution of *F*_ST_ across loci as a function of heterozygosity, reflecting migration–mutation–drift balance with no selection. Outlier loci correspond to those SNPs with observed *F*_ST_ values falling outside the 95% confidence interval (CI) neutral envelope (Beaumont & Nichols, 1996; Excoffier et al., 2009).

#### 2.5.2 Genome–environment association (GEA) outlier detection

We integrated two approaches to identify GEA outlier loci displaying significant statistical associations between allele frequency and climate variables. Bayesian generalized linear mixed models (BayEnv, Coop et al., 2010) relies on a Bayesian approach to test whether environmental factors improve fit over a null model (Günther & Coop, 2013). First, the non‐*F*_ST_ outlier SNP subset was used to estimate the null model of allele frequencies covariance across populations. Second, a Bayes factor (BF) is calculated for each SNP, representing the ratio of probabilities of a correlation between the allele frequency variation in the SNP and an environmental factor versus the null model given by the covariance matrix alone. Ten independent runs of 10^5^ MCMC cycles were produced for each SNP, and the means of the results were used to estimate correlations between allele frequencies and environmental variables. SNPs with log_10_ (BF) >0.5 have strong support for associations between allele frequencies and environmental gradients (Jeffreys, 1998).

Latent factor mixed models (LFMMs) can improve detection accuracy using latent variables and taking the population structure into account (Frichot et al., 2013). Ten independent runs with 10^5^ iterations after a 5 × 10^4^ burn‐in step were produced to compute correlations between allele frequencies and climate variables in “LEA” R package (Frichot & François, 2015). The latent factors were set to two for analyses including all populations and one for analyses within each lineage (Tibet and HDM‐WSP). SNPs with *p*‐values < .05 are significantly associated with climate.

#### 2.5.3 Multivariate relationship between genetic structure and environmental gradients

##### Linear relationships

Mantel tests of isolation by distance (IBD) and isolation by environment (IBE) were performed to assess linear relationships between geographic distance (IBD; pairwise Euclidean distance) or environmental distance (IBE; Bray–Curtis distance) and genetic distance (pairwise *F*_ST_) using R package “ecodist” (Goslee & Urban, 2007). To disentangle the effect of IBD and IBE, a partial Mantel test was used to measure IBD by controlling the impact of environment and IBE by controlling the impact of geography. In addition, multiple regression on distance matrix (MRM) analysis was performed to test a multiple regression of genetic distance on geographic and environmental distances. Statistical significance of Mantel tests and MRM was evaluated from 10^4^ permutations.

Redundancy analyses (RDAs) and partial redundancy analyses (*p*RDAs) were used to detect linear relationships between genetic variations (from SNPs and SSRs datasets) and multivariate climatic gradients, using “vegan” R package (Oksanen et al., 2017). SSRs and SNPs with the four climate variables were considered as constrained factors and geographic variables (longitude and latitude) as conditioned factors. Statistical significance was evaluated from 999 permutations.

##### Nonlinear relationships

Generalized dissimilarity modeling (GDM, Ferrier, Manion, Elith, & Richardson, 2007) was performed using the “gdm” package (Ferrier et al., 2007) to identify nonlinear relationships between genetic distance and environmental and geographic distances by fitting splines (Fitzpatrick & Keller, 2015). Geographic distance was based on Euclidean distance between sites, and the matrices of genetic distance were based on the mean allele frequency of each SNP. Spline shape and height describe the allelic compositional change along the environmental gradient and the importance of the environmental variable, respectively.

### 2.6 Risk of nonadaptedness under future climatic scenarios

We performed a risk of nonadaptedness (RONA, Rellstab et al., 2016) to predict the adaptive potential of *Q. aquifolioides* to future local climate using default settings in PYRONA v0.1.3 (Pina‐Martins, Baptista, Pappas, & Paulo, 2018). Briefly, the algorithm establishes marker–environmental associations, based on current allele frequencies and modern‐day environmental variables. The inferred linear model is used to predict expected allele frequencies in future environmental gradients in 2050 and 2070 according to the Global Climate Model BCC‐CSM1‐1 (IPCC, 2014) under two contrasted representative concentration pathways (RCPs), a low‐emission scenario (RCP26), and a high‐emission scenario (RCP85) based on regression curve. The "RONA value" is the mean difference between current and future expected allele frequencies, which estimates the average change in allele frequency that will be required for the populations to adapt to future climate.

## 3 RESULTS

### 3.1 Genetic structure and diversity

The 65 polymorphic candidate gene fragment sequences have been deposited in GenBank under accession numbers MH001439–MH001440, MH061101–MH061143, MH136995–MH137035, MH151806–MH151852, MH194560–MH194566, and MH210611–MH210644 (Table S2). Of the 65 successfully amplified polymorphic loci, 64 had at least one blast hit against *Q. robur* amino acid sequences (Table S3). We identified 71 coding sequences (CDSs) from 60 loci (Table S4). From the 51,623,432 paired‐end Illumina reads obtained from six sequencing libraries, 831 high‐quality biallelic SNPs were called by the GATK pipeline. Annotation of all SNPs is provided in Table S4. After quality filtering, we retained 381 SNPs, of which 355 and 378 were found in the Tibet and HDM‐WSP lineages, respectively (Table S5). Of the 381 SNPs, 207 (54%) resided in coding regions including 114 (55%) synonymous, 89 (43%) missense, and four (1.9%) stop‐gained variants (Table S5).

**FIGURE 2:**
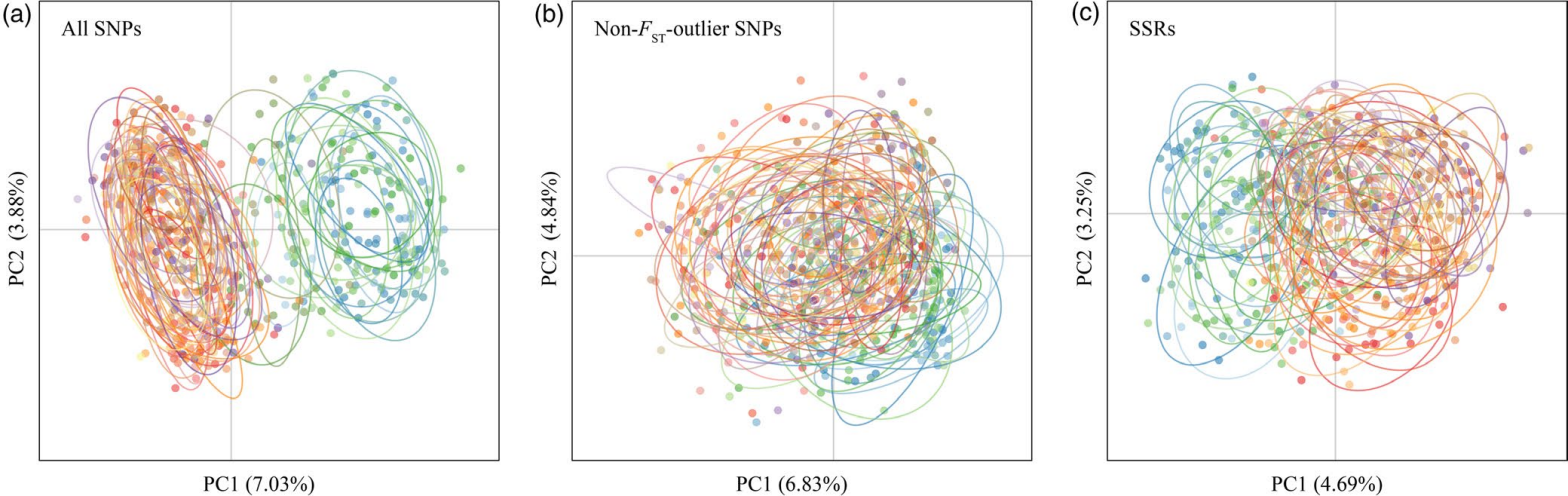
Principal component analysis based on (a) all SNPs, (b) non‐*F*_ST_ outlier SNPs, and (c) SSRs. Cool color dots (blue/green) illustrate Tibet lineage, and warmer color dots (red/orange) represent HDM‐WSP lineage

Principal component analysis using all SNPs clearly assigned the 587 sampled individuals to two distinct genetic lineages (Tibet and HDM‐WSP) that could not be distinguished on the basis of neutral markers (non‐*F*_ST_ outlier SNP subset or SSRs) alone (Figure 2). Using all SNPs, AMOVA indicated 13% of the molecular variance is explained by grouping populations in the two lineages (Table 1). Overall, based on non‐*F*_ST_ outlier SNPs and especially SSRs, diversity indices indicate lower level of genetic diversity in Tibet than in HDM‐WSP (most *p*‐values ≤ .05; Table 2, Table S6).

**TABLE 1:** Hierarchical analyses of molecular variance (AMOVA) of *Q. aquifolioides* populations based on all SNPs, non‐*F*_ST_ outlier SNPs, and SSRs

	All SNPs		Non‐*F*_ST_ outlier SNPs		SSRs	
	Percentage of variation (%)	Fixation indices	Percentage of variation (%)	Fixation indices	Percentage of variation (%)	Fixation indices
Tibet lineage						
Among populations	4.6	*F*_ST_ = 0.05	2.9	*F*_ST_ = 0.03	3.4	*F*_ST_ = 0.03
Within populations	95.4		97.1		96.6	
HDM‐WSP lineage						
Among populations	7.0	*F*_ST_ = 0.07	5.4	*F*_ST_ = 0.05	5.4	*F*_ST_ = 0.05
Within populations	93.0		94.6		94.6	
All populations						
Among lineages	13.4	*F*_CT_ = 0.13	6.4	*F*_CT_ = 0.06	4.6	*F*_CT_ = 0.05
Among populations within lineages	5.6	*F*_SC_ = 0.06	4.3	*F*_SC_ = 0.05	4.7	*F*_SC_ = 0.05
Within populations	81.1	*F*_ST_ = 0.19	89.3	*F*_ST_ = 0.11	90.7	*F*_ST_ = 0.09

Note

Significance tests (1,000 permutations) showed all fixation indices were significant (*p* < .001).

**TABLE 2:** Genetic diversity estimates for the investigated *Q. aquifolioides* populations. (a) Estimates based on all SNPs and non‐*F*_ST_ outlier SNPs. (b) Estimates based on SSRs

(a)					
Pop	*N*	*P*	*H*_O_	*H*_E_	*π*
All SNPs					
Tibet lineage	168	0.86	0.11	0.19	0.20
HDM‐WSP lineage	419	0.86	0.12	0.19	0.20
All populations	587	0.86	0.11	0.19	0.20
Non‐*F*_ST_ outlier SNPs					
Tibet lineage	168	0.86	**0.05^a^**	**0.12^a^**	**0.13^a^**
HDM‐WSP lineage	419	0.87	**0.07^b^**	**0.14^b^**	**0.15^b^**
All populations	587	0.87	0.06	0.14	0.15

(b)					
Pop	*N*	*N*_E_	*H*_O_	*H*_E_	*I*
Tibet lineage	168	**3.37^a^**	**0.71^a^**	**0.62^a^**	**1.27^a^**
HDM‐WSP lineage	419	**4.59^b^**	**0.75^b^**	**0.73^b^**	**1.62^b^**
All populations	587	4.23	0.74	0.70	1.51

Note

Indices in boldface followed by different superscript letters have statistically significant different values between the two lineages (*p*‐value ≤ .05).

Abbreviations: *H*_E_, mean expected heterozygosity; *H*_O_, mean observed heterozygosity; *I*, mean Shannon index; *N*, number of individuals; *N*_E_, mean size of effective population; *P*, mean frequency of the most frequent allele at each locus; *π*, mean nucleotide diversity.

### 3.2 *F*_ST_ outlier detection

**FIGURE 3:**
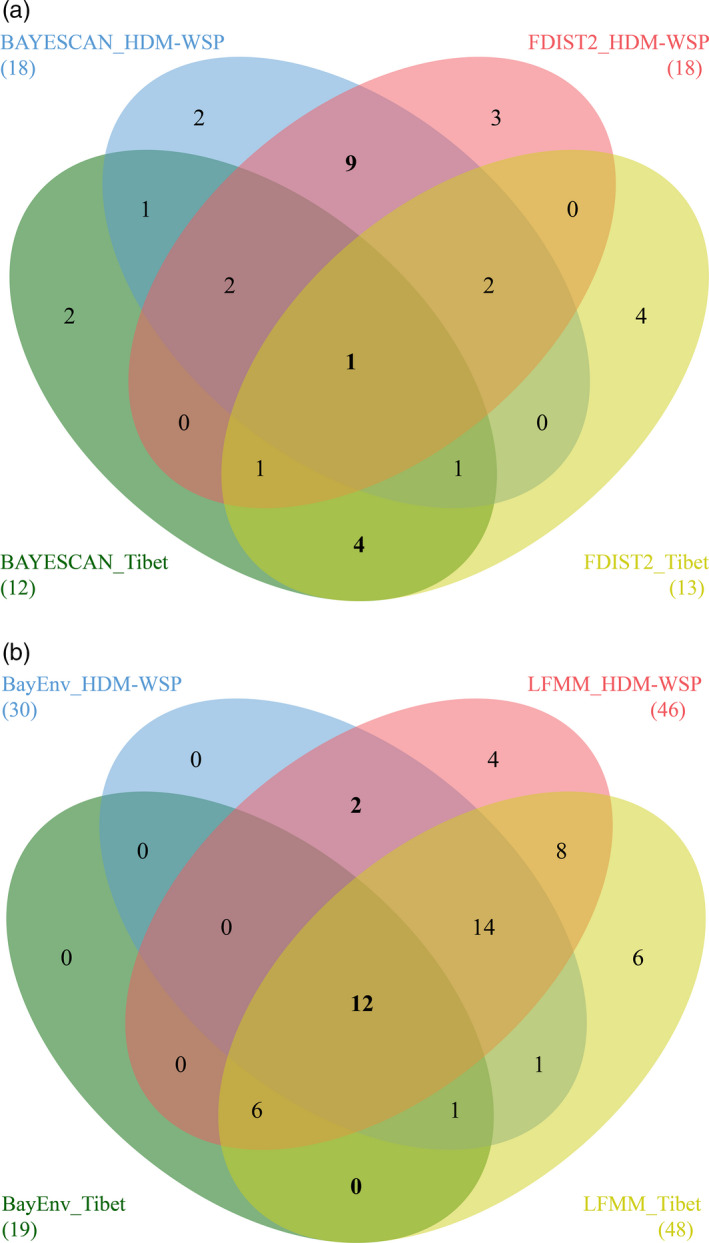
Venn diagrams showing genes containing outlier SNPs detected in the Tibet and HDM‐WSP lineages by (a) BAYESCAN and FDIST2 (i.e., *F*_ST_ outlier SNPs) and (b) BayEnv and LFMM (i.e., GEA outlier SNPs). The number of genes is given in each area, and the total number of genes for each lineage using each method is given in parentheses. The common and lineage‐specific outlier genes are in bold

Details of *F*_ST_ outlier SNPs are shown in Figure 3a, Figure S2 and Tables [Link], [Link]. In Tibet, seven candidate genes contained positive *F*_ST_ outlier SNPs according to both BAYESCAN and FDIST2 and four of these were lineage‐specific. By contrast, in HDM‐WSP, 14 candidate genes contained *F*_ST_ outlier SNPs and nine of them were unique to the lineage. Only one candidate gene containing *F*_ST_ outlier SNPs flagged by both methods was common to Tibet and HDM‐WSP (Figure 3a, Table S7).

### 3.3 GEA outlier detection

Details of GEA outlier SNPs are shown in Figure 3b, Figures S3 and S4, and Tables S7, S8 and S10. In the Tibet lineage, 19 candidate genes containing GEA outlier SNPs were detected by both BayEnv and LFMM, but none of them was unique to the region. In HDM‐WSP, 28 candidate genes contained GEA outlier SNPs and two of these genes (including three GEA outlier SNPs) were lineage‐specific (CL6004CT6724_02: multicopper oxidase and CL9715CT14526_03: long‐chain acyl‐CoA synthetase 4, LACS4; Figure S5). In total, 12 candidate genes containing GEA outlier SNPs were detected by both GEA approaches in the two lineages (Figure 3b; Table S7). Among these, 28 SNPs were significantly associated with the same climatic variable (prec01), and the remainder were associated with the other three climate variables (bio03, bio09 and prec06) (Table S7).

### 3.4 Linear relationships

**FIGURE 4:**
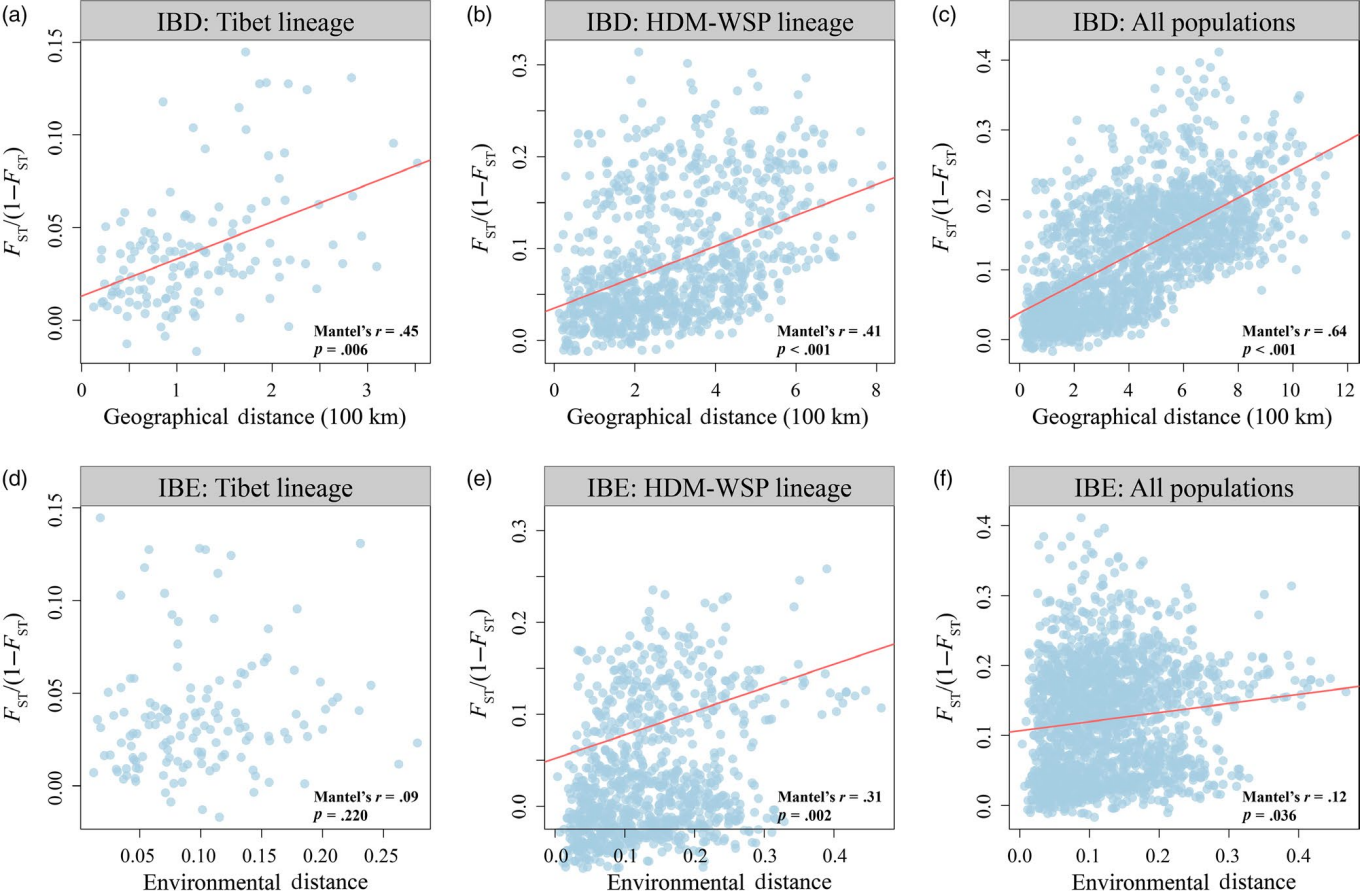
Mantel tests of genetic distance (*F*_ST_/(1 − *F*_ST_)) against geographic and environmental distances in each lineage and for all populations combined

Mantel and partial Mantel tests revealed spatial genetic structure consistent with IBD in Tibet and HDM‐WSP as well as for all populations combined. Correlation between genetic and environmental distances, consistent with IBE, was detected in HDM‐WSP and for all populations combined, but not in Tibet (Figure 4; Table S11). More specifically, genetic distance was significantly associated with precipitation during the dry season (prec01) in HDM‐WSP and for all populations (Table S12). These findings were corroborated by optimal MRM models that yielded similar results (Table S13).

The percentages of variance explained by RDA and *p*RDA were similar, and we thus report results for *p*RDA. In Tibet, SNP variation was not associated with geography or climate variables, whereas in HDM‐WSP and for all populations, temperature during the driest quarter (bio09) and precipitation during the dry season (prec01) contributed the most to SNP variation (Table 3; Figure S6). In HDM‐WSP, 11% of the SNP variance was explained by climate, while geography only accounted for 6%. For all populations combined, 8% of the explained SNP variance was due to climate and 9% to geography (Table 3). For the SSRs, *p*RDA revealed that precipitation during the dry season (prec01) explained most genetic variance in Tibet. In HDM‐WSP and all populations, prec06 and prec01 were the two environmental variables most explanatory, respectively (Table 3; Figure S7). Partitioning of the total SSR variance revealed that 5%, 3%, and 2% of the explained variance were due to climate, while 3%, 2%, and 4% were due to geography in Tibet, HDM‐WSP, and all populations, respectively (Table 3).

**TABLE 3:** Summary and partitioning of the variance associated with climate and geographic variables based on redundancy analysis (RDA) and partial RDA (*p*RDA) in the Tibet lineage, the HDM‐WSP lineage, and all populations combined for SNPs and SSRs

	SNPs			SSRs		
	PVE	Eigenvalue	*P*	PVE	Eigenvalue	*P*
Tibet lineage						
Climate	23.62/22.79	24.58/23.72	0.814/0.849	6.35/5.18	0.66/0.54	0.001/0.001
Geography	11.32	11.78	0.736	2.88	0.30	0.001
bio03	4.36/4.96		0.985/0.897	1.76/1.33		0.001/0.001
bio09	7.12/7.32		0.227/0.290	1.90/1.77		0.001/0.001
prec01	5.10/4.14		0.901/0.964	0.71/1.91		0.177/0.002
prec06	7.04/6.37		0.263/0.532	1.99/0.88		0.001/0.032
HDM‐WSP lineage						
Climate	11.80/11.10	13.68/12.87	0.001/0.005	3.60/2.98	0.51/0.42	0.001/0.001
Geography	6.30	7.30	0.004	2.10	0.30	0.001
bio03	2.40/2.44		0.389/0.298	0.50/0.66		0.001/0.001
bio09	3.37/3.03		0.004/0.019	0.84/0.68		0.001/0.001
prec01	3.12/2.85		0.017/0.060	0.95/0.69		0.001/0.001
prec06	2.92/2.76		0.045/0.082	1.31/0.95		0.001/0.001
All populations						
Climate	8.20/7.77	9.84/9.34	0.014/0.003	2.96/2.45	0.40/0.33	0.001/0.001
Geography	8.87	10.66	0.001	4.44	0.60	0.001
bio03	1.57/1.74		0.636/0.213	0.49/0.39		0.001/0.001
bio09	2.39/2.16		0.016/0.014	0.64/0.61		0.001/0.001
prec01	2.30/1.80		0.021/0.136	1.50/0.79		0.001/0.001
prec06	1.94/2.08		0.165/0.020	0.78/0.67		0.001/0.001

Note

The values on the left and right sides of the slash represent the RDA and *p*RDA results, respectively.

Abbreviations: PVE, percentage of explained variance.

### 3.5 Nonlinear relationships

**FIGURE 5:**
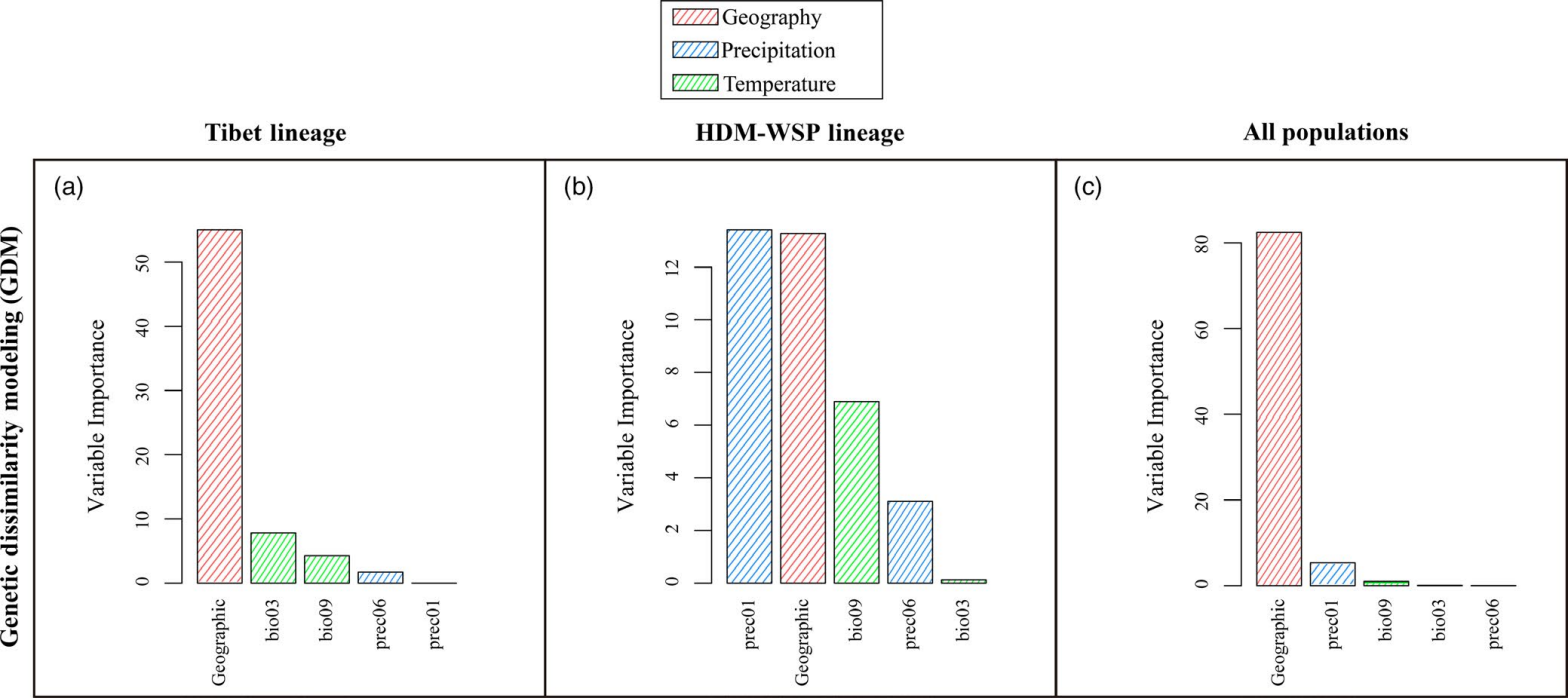
Ranked importance of environmental variables based on analysis of a generalized dissimilarity model (GDM) for each lineage and for all populations combined. Exact values are shown in Table S14

Geographic and environmental distances for the variables considered with GDM explained 82% and 6% of genetic variation for all populations, 55% and 14% of variation in Tibet, and 13% and 24% in HDM‐WSP (Table S14). Hence, while geography was the most important predictor in Tibet and for all populations combined, prec01 was the most important environmental factor related to genetic differentiation in HDM‐WSP lineage (Figure 5). These results were corroborated by I‐spline analysis (Figures S8–S10).

### 3.6 Risk of nonadaptedness under future climatic scenarios

**FIGURE 6:**
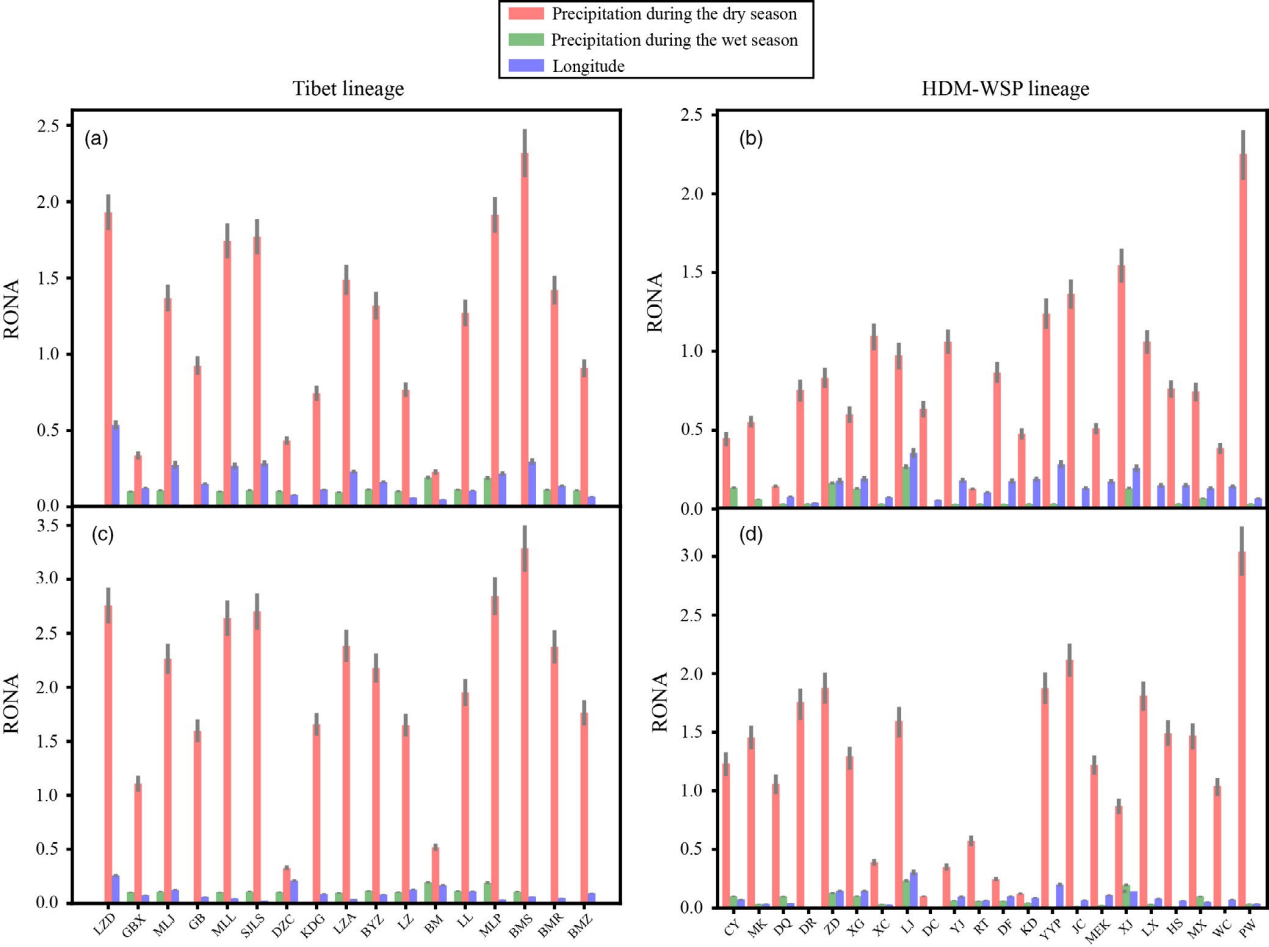
Risk of nonadaptedness plot (RONA) for three environmental factors under RCP26 (top panels) and RCP 85 (lower panels) prediction models in 2070. Bars represent weighted means (by *R* ^2^ value), and lines represent standard error for each population in the Tibet and HDM‐WSP lineages. The exact *R* ^2^ values are shown in Table S16

The most represented environmental factor identified by RONA analyses was the precipitation during the dry season (prec01) in Tibet and HDM‐WSP lineages. The RONA value of Tibet lineage was on average higher than that of HDM‐WSP lineage under both RCP26 and RCP85 predictions in 2050 and 2070 (Figure 6; Figures S11 and S12 and Tables S15 and S16), indicating that the HDM‐WSP lineage has higher potential to adapt to future local climate than Tibet lineage. Interestingly, peripheral population PW located at the easternmost end of the Hengduan Mountains had a lower adaptedness potential for prec01 compared with all other populations in HDM‐WSP lineage, indicating that this marginal *Q. aquifolioides* population isolated at the eastern edge of the distribution might be at higher risk of extinction under future climate. The RONA value in this peripheral population is similar to that of the most vulnerable populations in Tibet lineage (e.g., BMS, MLP).

## 4 DISCUSSION

Our study integrated numerous population genetics and landscape genomic methods to detect putative adaptive genetic variation in *Q. aquifolioides* populations throughout the species' range in the Himalaya–Hengduan Mountains. We first relied on *F*_ST_ outlier and GEA methods to reveal SNP markers consistent with locus‐specific imprints of natural selection in each lineage individually and throughout the species range. Based on annotations of these potentially adaptive SNP variants and their predicted coding effects, we can compare the functional differences between genes identified in the distinct lineages and further analyze the effects of these differences on the ecological adaptation of *Q*. *aquifolioides* in different lineages. We also performed linear and nonlinear multivariate analyses by integrating genetic and environmental factors to identify complex relationships between SNP frequency gradients with climate variables and determine key climate variables most likely to trigger adaptive response in *Q. aquifolioides*. We finally predicted the capacity of *Q. aquifolioides* to adapt to future local climate, based on two modeled scenarios of climate warming.

We found that genetic variation in *Q. aquifolioides* showed contrasted patterns of local adaptation in the two lineages. In Tibet lineage, the genetic variation was mainly driven by geographical distance, whereas in HDM‐WSP lineage, the climatic variables, especially precipitation, were instrumental in shaping a genetic imprint consistent with adaptive genetic variation.

### 4.1 Patterns of genetic differentiation and diversity in *Q. aquifolioides*

Results from AMOVA and PCA revealed higher level of genetic differentiation when all SNPs were considered compared with reduced marker datasets including only non‐*F*_ST_ outlier SNPs or SSRs. Diversifying selection can reduce effective gene flow and increase divergence across populations (Guichoux et al., 2013; Nosil, Funk, & Ortiz‐Barrientos, 2009). In addition, gene flow across oak populations is limited due to the complex topography in the Himalaya–Hengduan Mountains (Meng et al., 2017), and hence, local adaptation in *Q. aquifolioides* should not be prevented by intraspecific gene flow or possible introgression with other sympatric oak species (i.e., to date, there is no clear evidence of introgression). Higher *F*_ST_ values reported for analyses including all SNPs suggested that at least some of the positive *F*_ST_ outlier loci could have been affected by diversifying selection among homogenous gene pools (or be in linkage disequilibrium with such loci). Another explanation for the higher *F*_ST_ values is that random genetic drift eventually caused fixation of different alleles in each lineage due to limited gene flow. However, this seems unlikely because GEAs suggest some gene SNPs are actually related to environmental variables, a genomic imprint also consistent with local adaptation.

The higher level of genetic diversity along with a greater proportion of private alleles in the HDM‐WSP lineage than in the Tibet lineage reflects the demographic history of the species inferred from chloroplast DNA and microsatellite markers (Du et al., 2017), which suggests that the HDM‐WSP lineage received more immigrants, likely acted as a refugium, and plays an important role for the evolution and the maintenance of species diversity (Favre et al., 2015; López‐Pujol, Zhang, Sun, Ying, & Ge, 2011). The reduced genetic diversity with fewer private alleles in the Tibet lineage likely reflects extinction–recolonization dynamics in this area, whereby only a subset of the Hengduan Mountain refugium gene pool recolonized Tibet (Du et al., 2017; Meng et al., 2017; Qiu, Fu, & Comes, 2011).

### 4.2 Functional interpretation of outlier candidate gene SNPs

Accurately identifying loci most likely to be under selection is a crucial step in candidate gene studies. All methods present their own limitations and advantages (de Lafontaine et al., 2018; Hoban et al., 2016; Sork, 2018). In the present study, a combination of *F*_ST_‐based and genotype–environment association (GEA) methods was used, as this should increase the robustness of outlier detection. Another challenge is to find the most likely causes of outlier syndrome. In this respect, the recent release of the oak genome (Plomion et al., 2018) provides useful information to help interpreting gene functions. In addition, thanks to a large number of genomic studies focusing on drought stress in plants, our results can be contextualized in light of available knowledge about these stress‐related genes.

Genotype–environment associations identified 12 genes associated with climate variables (mostly precipitation during the dry season—prec01) in both Tibet and HDM‐WSP lineages, suggesting some level of parallel adaptation to drought in the species. Moreover, GEAs identified two genes displaying lineage‐specific association with climatic variables in HDM‐WSP (CL6004CT6724_02 and CL9715CT14526_03). Three SNPs within these two genes were associated with temperature during the driest quarter (bio09) and precipitation during the dry season (prec01). These three markers were also flagged as positive *F*_ST_ outlier SNPs in the HDM‐WSP lineage by both BayeScan and FDIST2 approaches, further suggesting that they might have evolved under divergent drought selective pressure in this lineage. One SNP is located on CL6004CT6724_02, a gene encoding metal oxidase, which is important in the physiological processes of plants, as it acts on plant growth, reproduction, and development in a variety of forms, including callus formation and lignin synthesis (de Tullio, Liso, & Arrigoni, 2004; Sanmartin, Pateraki, Chatzopoulou, & Kanellis, 2007). The other two SNPs are located on CL9715CT14526_03 gene (Table S4). This gene encodes long‐chain acyl‐CoA synthetase 4 (*LACS4*) that plays an important role in the anabolism and catabolism of fatty acids. Fatty acids are the basic building blocks of most lipids, including waxes and cutin important for protecting plants from biotic and abiotic stresses (Shockey & Browse, 2011). In *Arabidopsis*, *LACS4* inactivation resulted in a significant overaccumulation of tryphine lipids and displayed morphological anomalies of the pollen grains (Jessen et al., 2011). Moreover, stem of *lacs4* mutants showed significantly reduced total wax levels relative to the wild type (Jessen et al., 2011). The variation in these three SNPs may affect the regulatory expression of genes, further affecting the HDM‐WSP lineage's ability to adapt to key environmental factors related to dry environments (bio09 and prec01). This also indicates the role of environmental factors as important selective pressure likely driving genetic variation and fostering local adaptation of the HDM‐WSP lineage. By contrast, GEAs did not reveal specific genes associated with environmental variables that are unique to the Tibet lineage, suggesting a different adaptation pattern compared with the HDM‐WSP lineage.

### 4.3 Contrasted patterns of genetic variation across the range

Mantel and partial Mantel tests as well as MRM analysis all revealed significant IBD patterns in the Tibet lineage and in all populations combined. These results were corroborated by GDM and RDA: In Tibet, geographic distance, not the environment, explained high variable importance and a high percentage of explained variance (PVE), respectively. Together, these results indicate that the genetic signature of the Tibet lineage mainly reflects demographic evolution, but not strong selective pressure from the environment.

By contrast, Mantel and partial Mantel tests as well as MRM analysis indicated significant IBE in the HDM‐WSP lineage. Accordingly, GDM and RDA both suggested precipitation during the dry season (prec01) was the most significant environmental factor driving genetic variation in the HDM‐WSP lineage. Furthermore, RONA analyses predicted that HDM‐WSP lineage should have a higher capacity to adapt compared with Tibet lineage in the face of projected climate warming. This result is also supported by the higher number of lineage‐specific outliers (both *F*_ST_ and GEA) in HDM‐WSP compared with Tibet, indicating HDM‐WSP lineage might include more potentially adaptive polymorphisms than Tibet. Hence, the Tibet lineage might be more vulnerable under future climate and will have to shift higher allele frequencies than HDM‐WSP lineage in order to persist locally. Relatively warm and humid climatic conditions experienced by populations of the HDM‐WSP lineage might have been instrumental for local adaptation, as reported in other organisms, including fishes (Hecht, Matala, Hess, & Narum, 2015), birds (Bay et al., 2018), and trees (Gugger, Liang, Sork, Hodgskiss, & Wright, 2018). Our findings indicate that the Tibet and HDM‐WSP lineages display contrasted patterns of adaptive genetic variation, with genetic diversity in Tibet being mostly shaped by demographic evolutionary forces and genetic diversity in the HDM‐WSP lineage likely reflecting a greater contribution of natural selection to drought stress. Many other studies have reported genetic patterns consistent with local adaptation to climate in *Fagus* (Csilléry et al., 2014; Pluess et al., 2016), oaks (Gugger et al., 2018; Pina‐Martins et al., 2018; Sork et al., 2016), and *Castanopsis* (Sun, Surget‐Groba, & Gao, 2016). Here, we investigated these patterns in two lineages and revealed strikingly contrasted signatures of evolutionary dynamics across the range.

## 5 CONCLUSION

Combining the results from population genetics and landscape genomic methods, we detected contrasted patterns of genetic variation in response to environmental gradients in *Quercus aquifolioides*, a naturally distributed forest species. Selectively neutral evolutionary processes (e.g., isolation by distance) are key drivers of the genetic variation in the Tibet lineage, whereas adaptive processes (e.g., isolation by environment) prevail for shaping genetic diversity of the HDM‐WSP lineage. More specifically, we identified two genes that were correlated with climate gradients only in the HDM‐WSP lineage, supporting lineage‐specific signals of local adaptation. Our study thus provides valuable insights on local adaptation in trees and their main environmental drivers. In the changing climatic environment associated with global warming, the HDM‐WSP lineage may provide valuable genetic resources for the species. In the near future, more studies integrating genomic, phenotypic, and environmental data are required to gain further insights into the mechanisms of oak adaptation.

## CONFLICT OF INTEREST

None declared.

## AUTHOR CONTRIBUTIONS

FD designed the research; TRW and YYW performed the experiments; FD, TRW, YYW, and SU performed the analysis; FD, SU, and GdL wrote the manuscript; and all authors revised the manuscript.

## Supporting information

Figure S1‐S12

Table S1‐S7

Table S8‐S16

## Data Availability

Data for this study are available at Dryad: https://doi.org/10.5061/dryad.f1vhhmgtk
