# Supplementary material for: Contrasted patterns of local adaptation to climate change across the range of an evergreen oak, Quercus aquifolioides
Source: Evol Appl. 2020 Jun 9;13(9):2377–91. doi: 10.1111/eva.13030 (PMC7513717; doi:10.1111/eva.13030)

**Figure S1.** Flow chart of pool-sequencing method. Paired-end barcodes were designed to separate sample individuals.


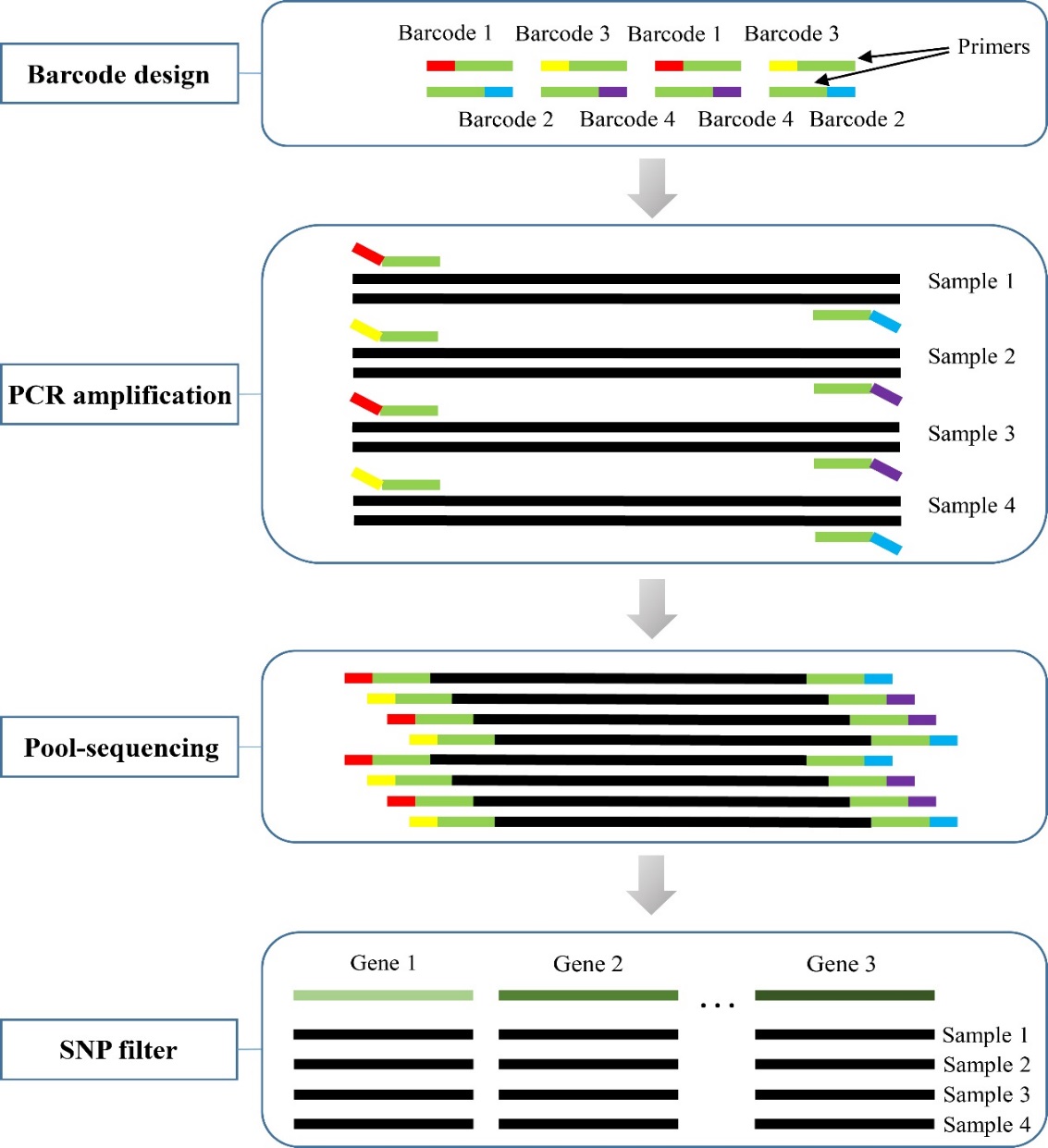


**FIGURE S2** Identification of *F*_ST_-outlier SNPs. (a-c) Distribution of observed *F*_ST_ values as a function of log_10_ (PO) obtained using BAYESCAN. The vertical lines indicate the log_10_ (PO) that corresponds to PO = 2. The dots to the right of the vertical lines represent the SNPs with outlier *F*_ST_ values. (d-f) Distribution of observed *F*_ST_ values as a function of heterozygosity obtained using FDIST2. The grey lines indicate the 2.5% and 97.5% boundaries of the simulated values. Dots outside the boundaries represent SNPs with outlier *F*_ST_ values. Detailed description of the outlier SNPs is presented in Table S7.


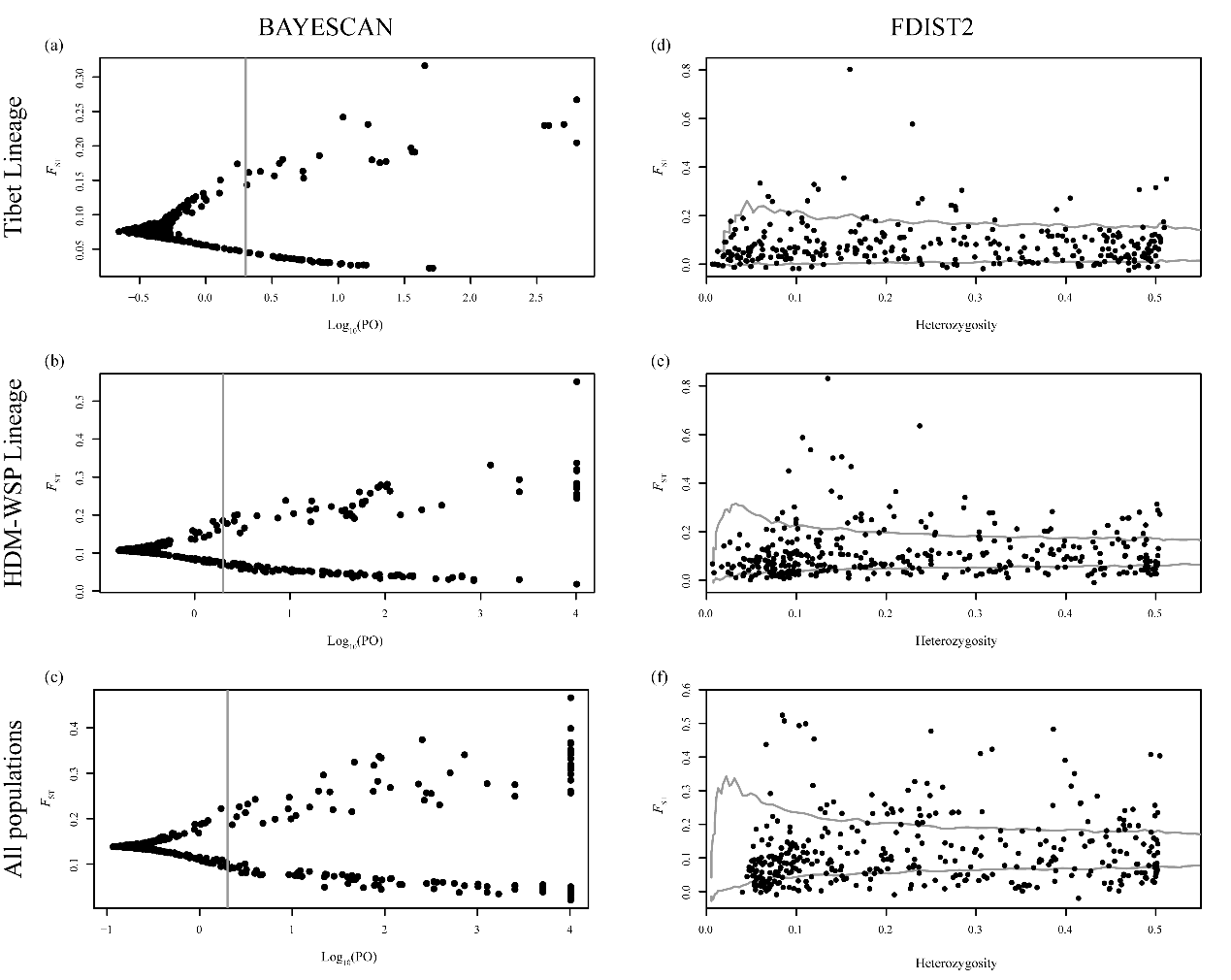


**Figure S3.** Manhattan plot of SNPs called from three scales with four environment variables by BayEnv. X-axis represents all SNPs and y-axis represents log_10_ (*Bayes Factor*) for each SNP.


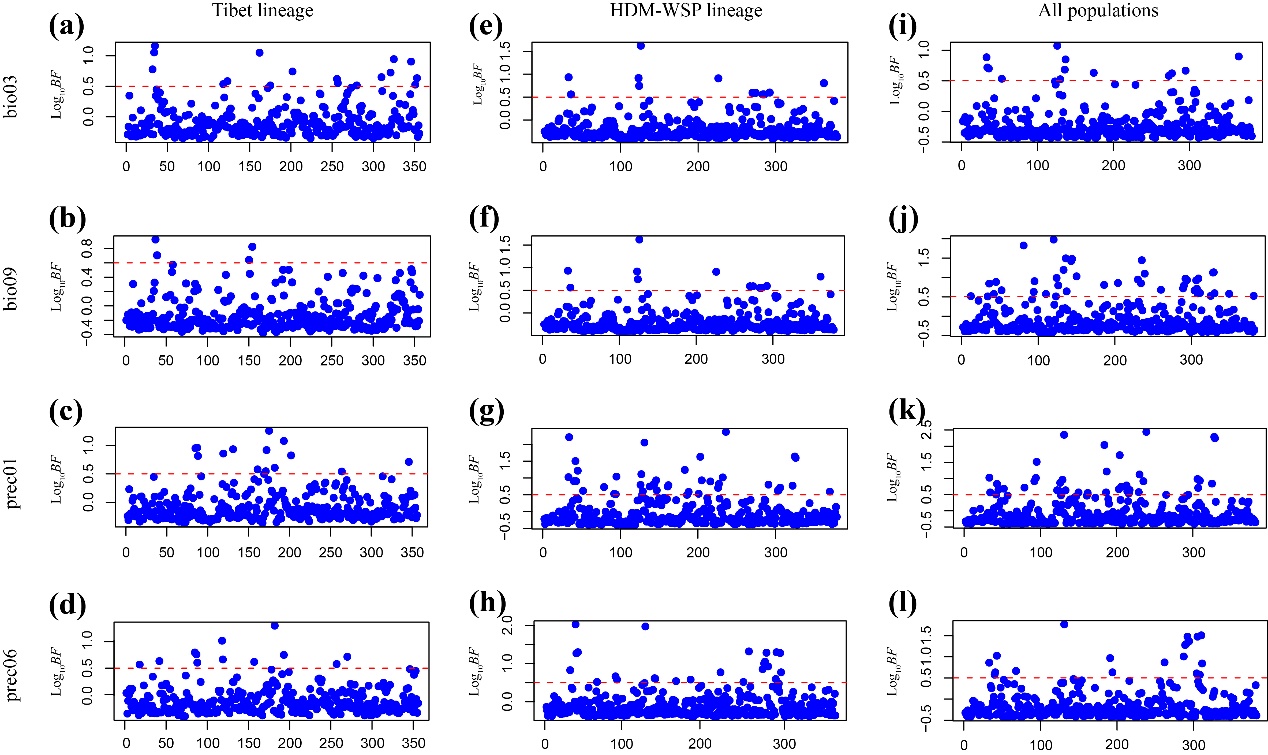


**Figure S4.** Manhattan plot of SNPs called from three scales with four environment variables by LFMM. X-axis represents all SNPs and y-axis represents -log_10_ *p*-value for each SNP.


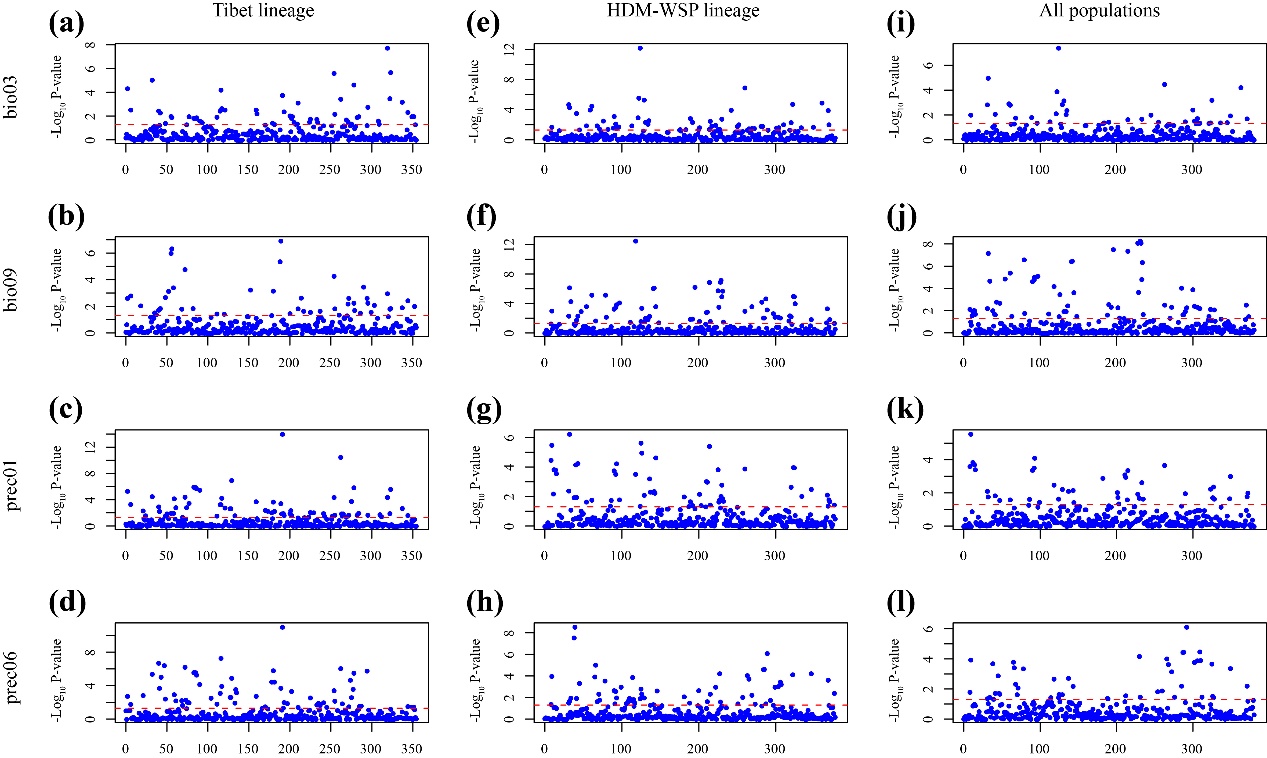


**Figure S5.** Schematic representation of SNPs on candidate genes in *Q. aquifolioides*.


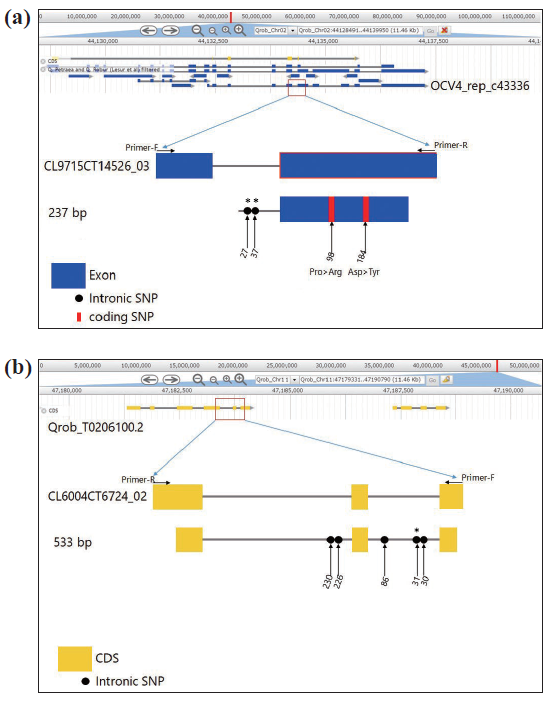


Jbrowse image for Oak genome assembly PM1N is shown at the top of each pannel, Primer-F and Primer-R are as in Table S2 for each gene and the expected amplicon is shown. The reference genomic sequence from Q. aquifolioides is aligned and SNP positions are indicated. (a) CL9715CT14526_03 gene: two SNPs (98, 184) belong to missense variant (Fig. S4; Table S7); two SNP (27, 37) located in upstream of the gene associated with mean temperature of the driest quarter (bio09) and precipitation during the dry season (prec01) based on BayEnv and LFMM approaches was indicated in asterisk (Table S5). The URL of the Jbrowse for Oak genome assembly PM1N is as follows: “https://urgi.versailles.inra.fr/WebApollo_oak_PM1N/jbrowse/?loc=Qrob_Chr02%3A44128491..44139950&tracks=DNA%2CAnnotations%2CGene_Release2.2_all_tag_polyp%2CQrob_PseudoMol_V2_Q_petreaAndQ_robur_addDiese&highlight=ghlight%3DQrob_Chr11%3A33871871..33880619”. Note: CL9715CT14526_03 gene SNP positions (184) was deleted in the filtering process of MAF. (b) CL6004CT6724_02 gene: three (30, 31, and 86) and two (226, and 230) SNPs located in downstream and upstream of the exon, respectively (Fig. S4; Table S7). One SNP position (31) associated with mean temperature of the driest quarter (bio09) based on BayEnv and LFMM approaches was indicated by the asterisk (Table S5). The URL of the Jbrowse for Oak genome assembly PM1N is as follows: “https://urgi.versailles.inra.fr/WebApollo_oak_PM1N/jbrowse/?loc=Qrob_Chr11%3A47179331..47190790&tracks=DNA%2CAnnotations%2CGene_Release2.2_all_tag_polyp&highlight=”.

**Figure S6.** Loadings of climate variables with first two axes based on SNPs.


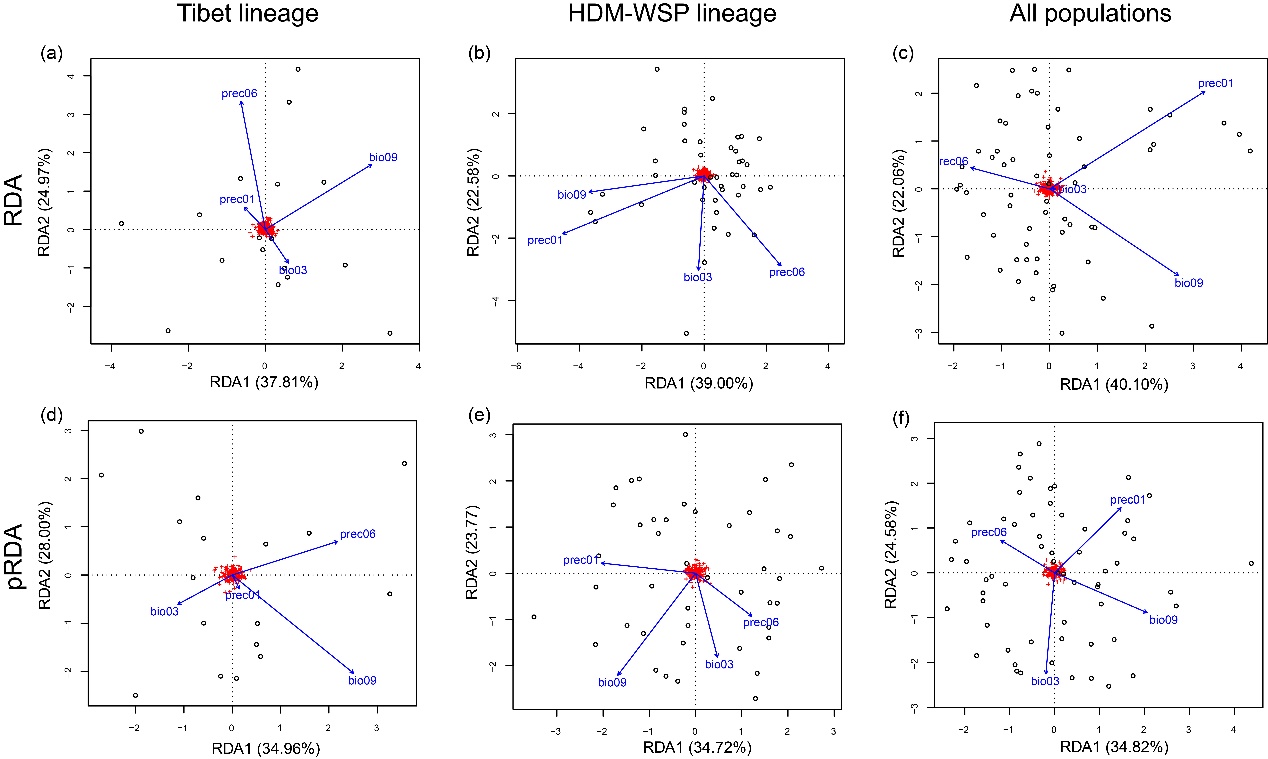


**Figure S7.** Loadings of climate variables with first two axes based on SSRs.


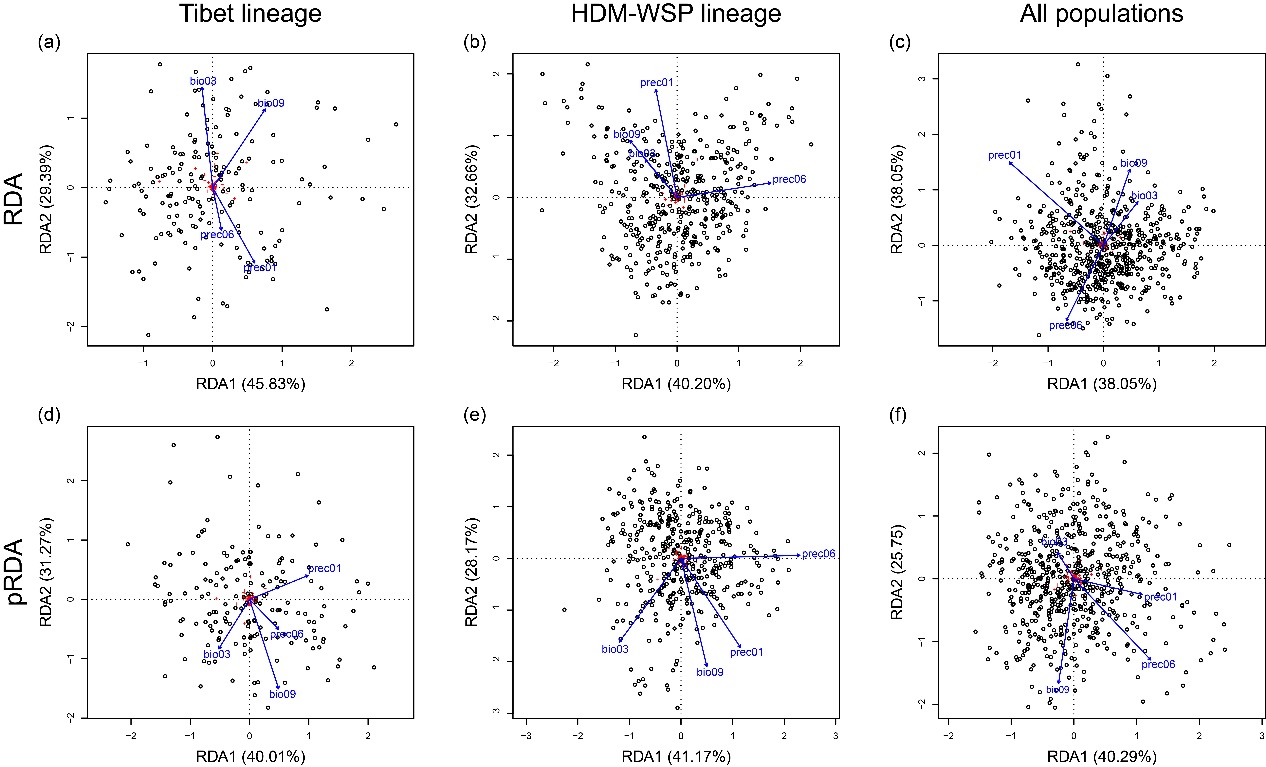


**Figure S8.** I-splines showing changes in genetic distance along geographic and environmental distance gradients in Tibet lineage (I-splines plot for prec01 is not shown because all coefficients were equal to zero).


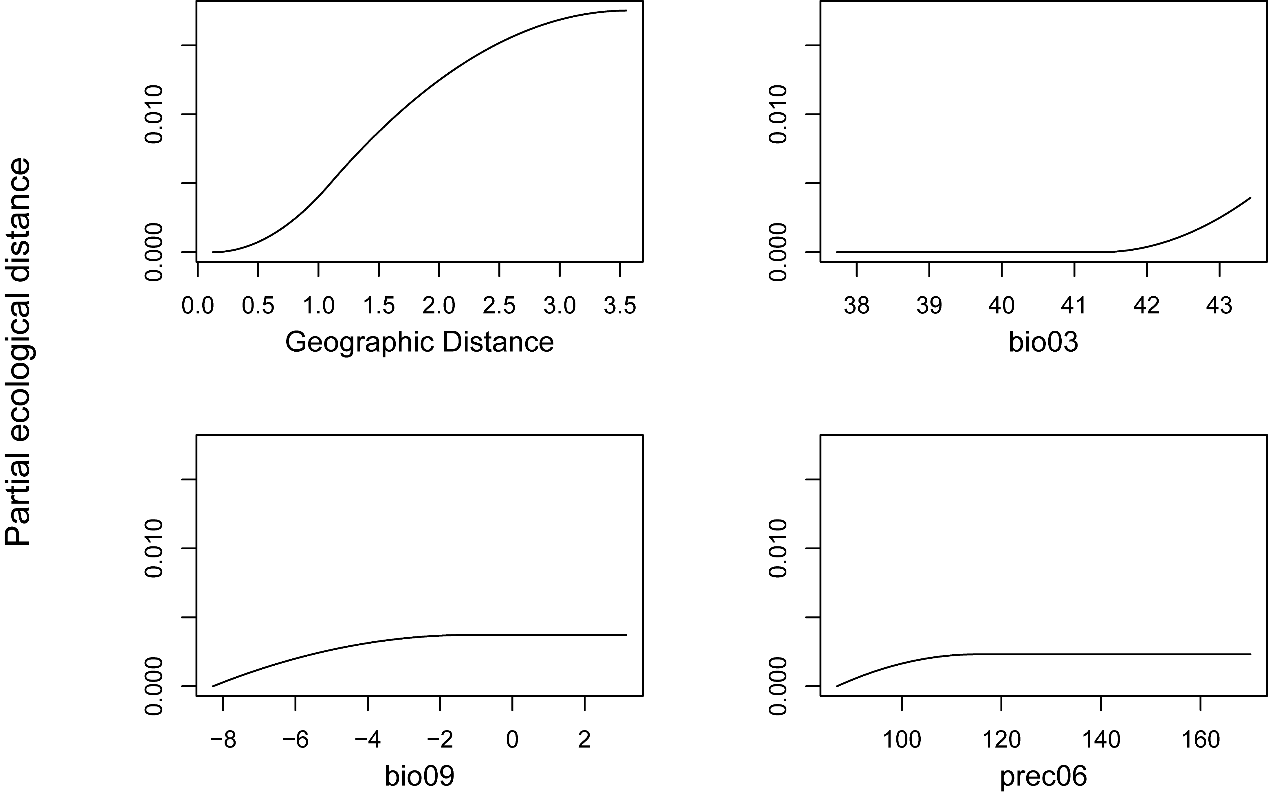


**Figure S9.** I-splines showing changes in genetic distance along geographic and environmental distance gradients in HDM-WSP lineage.


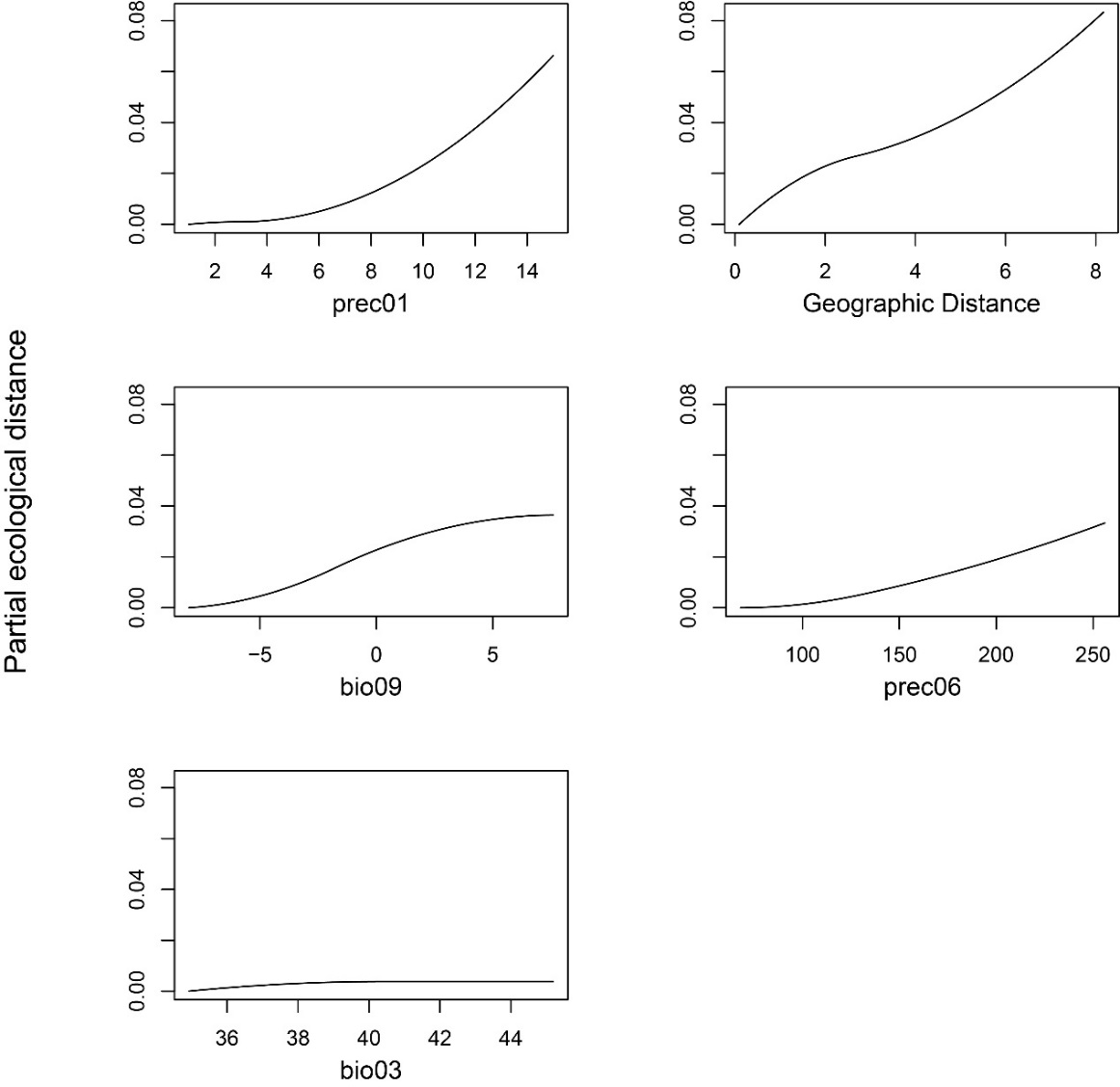


**Figure S10.** I-splines showing changes in genetic distance along geographic and environmental distance gradients in all populations.


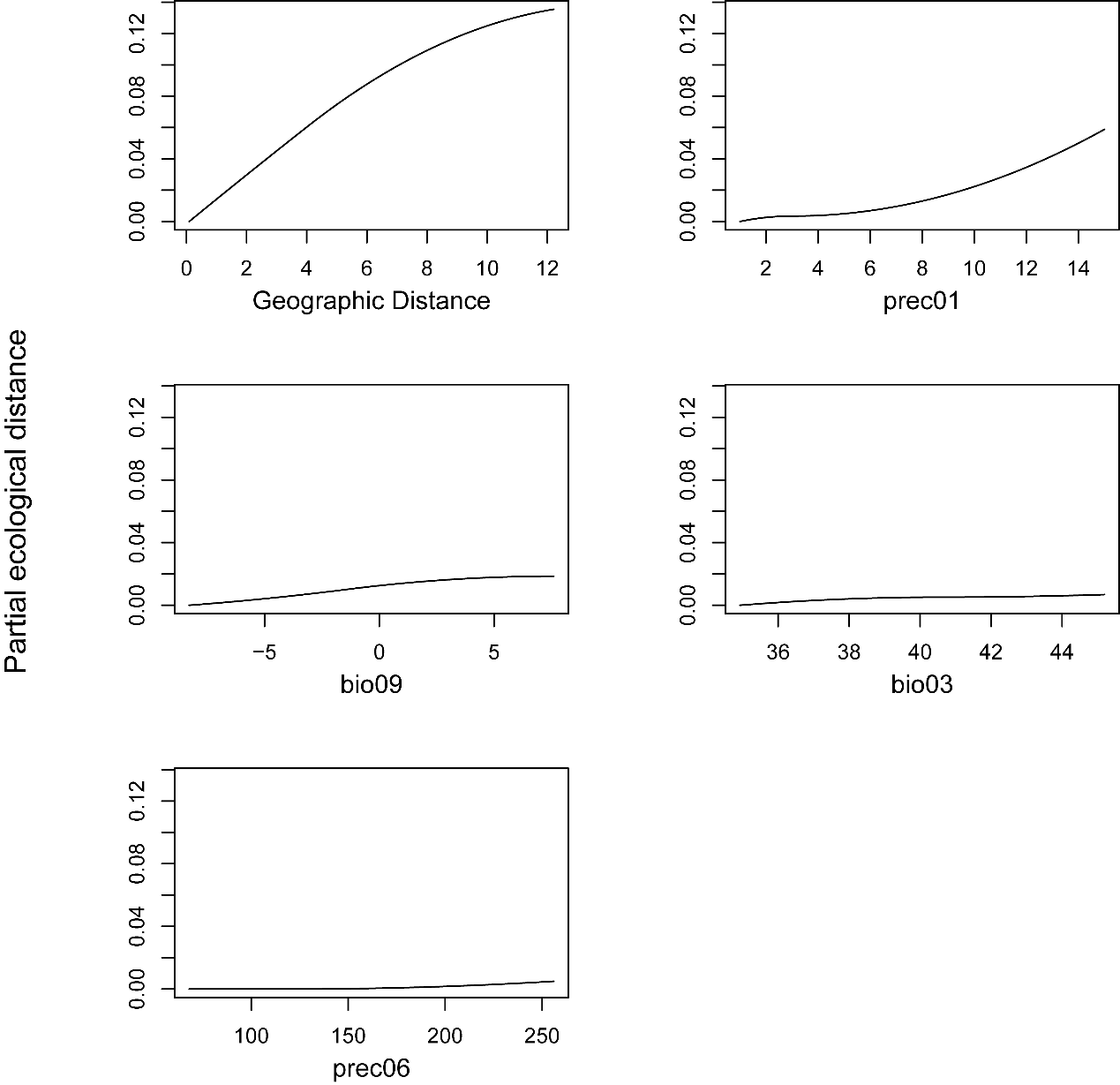


**Figure S11.** Risk of non-adaptedness (RONA) plot for three environmental factors under RCP26 prediction models in 2050. Bars represent weighted means (by R^2^ value) and lines represent standard error for each populations in the (a) Tibet and (b) HDM-WSP lineages. The exact R^2^ values are shown in Table S15.


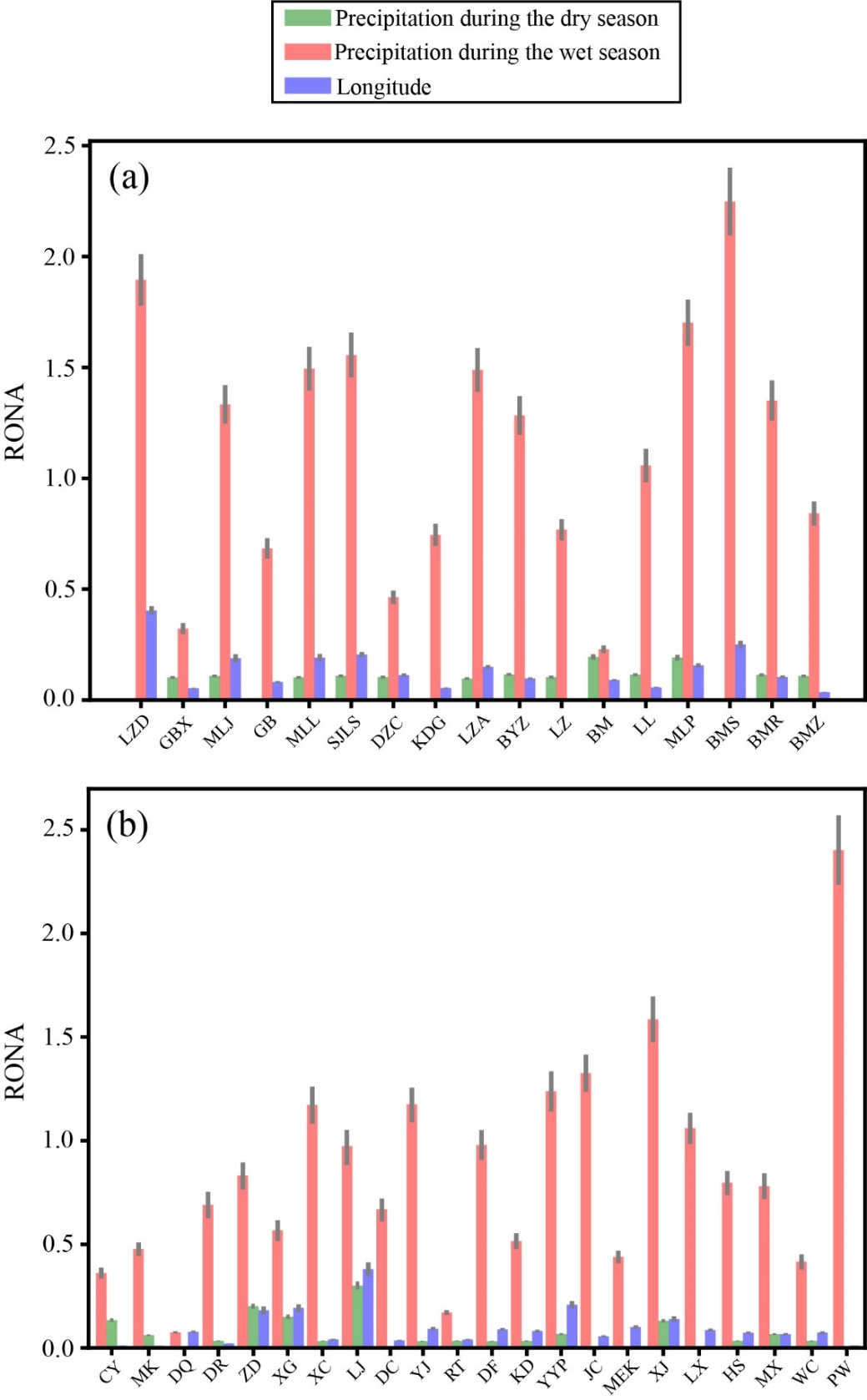


**Figure S12.** Risk of non-adaptedness (RONA) plot for three environmental factors under RCP85 prediction models in 2050. Bars represent weighted means (by R^2^ value) and lines represent standard error for each populations in the (a) Tibet and (b) HDM-WSP lineages. The exact R^2^ values are shown in Table S15.


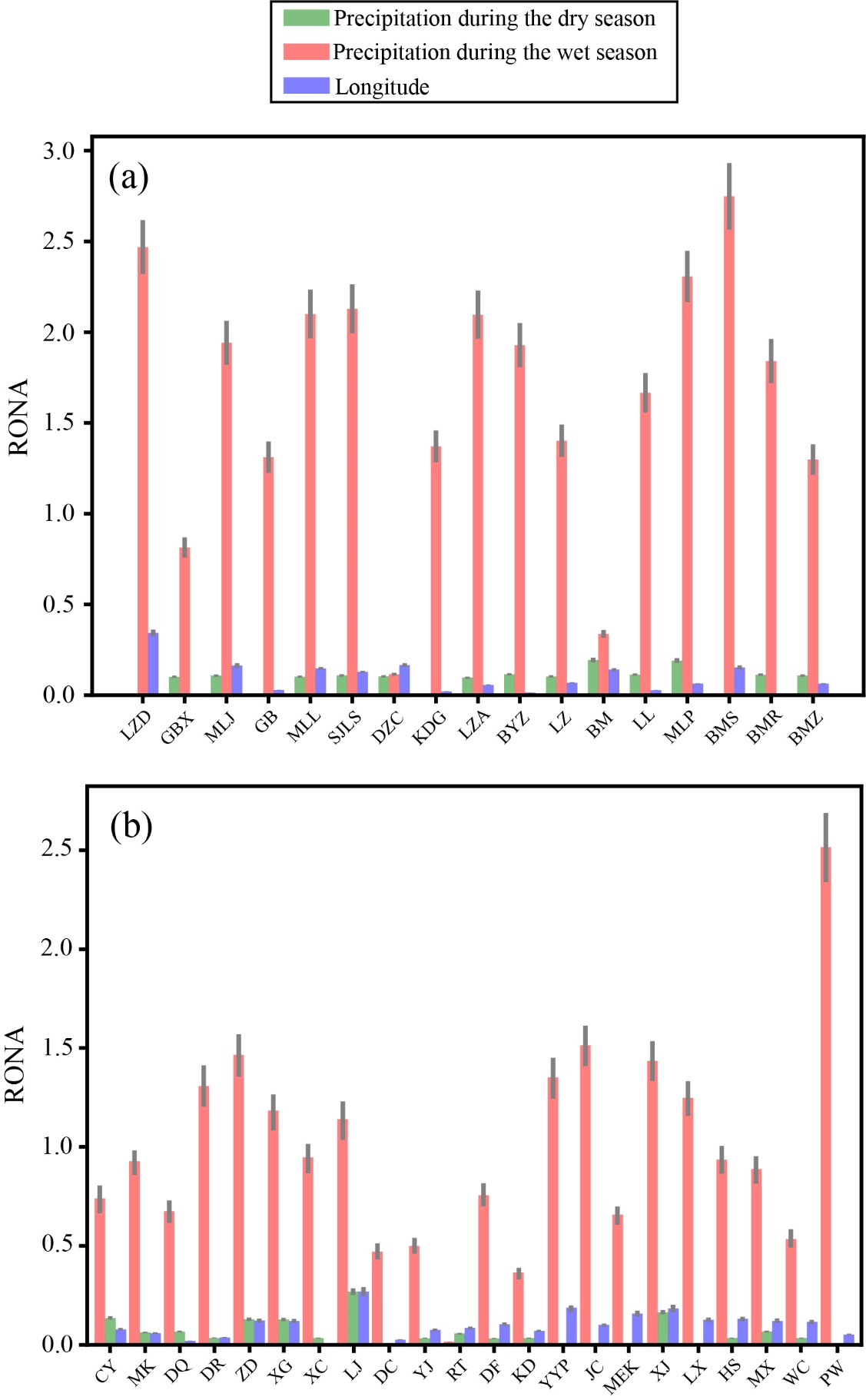

Supplement: Supplementary file 1 — Figure S1‐S12 [file EVA-13-2377-s001.docx]
