# Supplementary material for: Contrasted patterns of local adaptation to climate change across the range of an evergreen oak, Quercus aquifolioides
Source: Evol Appl. 2020 Jun 9;13(9):2377–91. doi: 10.1111/eva.13030 (PMC7513717; doi:10.1111/eva.13030)
Supplement: Supplementary file 3 — Table S8‐S16 [file EVA-13-2377-s003.docx]

**Table S8.** Number of outlier SNPs detected by each method. Corresponding number of outlier genes shown in parentheses.

|  | **BAYESCAN** | **FDIST2** | **BayEnv** | **LFMM** | **Total** |
| --- | --- | --- | --- | --- | --- |
| Tibet lineage | 23 (12) | 28 (13) | 39 (19) | 126 (48) | 148 (49) |
| HDM-WSP lineage | 53 (18) | 57 (18) | 83 (30) | 143 (46) | 180 (48) |
| All populations | 56 (24) | 67 (27) | 76 (32) | 123 (39) | 169 (45) |
| Total | 96 (33) | 104 (34) | 119 (38) | 206 (54) | 255 (54) |

**Table S9** List of common and lineage-specific *F*_ST_-outlier genes detected from Tibet and HDM-WSP lineage by both BAYESCAN and FDIST2.

| **Gene** | **Function** |
| --- | --- |
| *Common* |  |
| CL6811CT7609_01 | mitotic control protein dis3, putative |
| *Specific to HDM-WSP lineage* |  |
| CL4053CT4574_03 | AAP6 (AMINO ACID PERMEASE 6) |
| CL5359CT11480 | glutathione S-transferase |
| CL568CT709_01 | flavonoid 3-hydroxylase, putative |
| CL5734CT6423_01 | calcium dependent protein kinase |
| CL7115CT7941 | 4-coumarate:coA ligase 2 |
| CL8521CT9806_02 | 26S proteasome non-ATPase regulatory subunit |
| CL9629CT11162 | phosphoprotein phosphatase, putative |
| CL9715CT14526_03 | long-chain acyl-CoA synthetase 4 |
| WZ0AQRAQ4YD18FM1_02 | Myb-related transcription factor |
| *Specific to Tibet lineage* |  |
| CL7466CT8329_01 | GDH2 |
| CL8437CT9437_01 | 4-coumarate-CoA ligase |
| CL8450CT11779_02 | heat shock protein 70-3 |
| CL9715CT16564_04 | putative transaldolase |

**Table S10** List of common and lineage-specific GEAs outlier genes detected from Tibet and HDM-WSP lineage by both BayEnv and LFMM.

| **Gene** | **Function** |
| --- | --- |
| *Common* |  |
| CL3075CT3496 zinc finger protein | |
| CL5617CT6296_02 amino acid binding protein | |
| CL5734CT6423_01 calcium dependent protein | |
| CL6811CT7609_01 mitotic control protein dis3 | |
| CL7115CT7941 4-coumarate:coA ligase 2  CL3828CT4328 Xylem serine proteinase 1 precursor  CL4642CT5216 NAC domain protein NAC4  CL5719CT6408_02 indole-3-acetic acid 14 transcription factor  CL7021CT7837_01 abscisic acid responsive elements-binding protein 2  CL8464CT12114_01 glutaredoxin  CL8521CT9806_02 26S proteasome non-ATPase regulatory subunit  CL9715CT16564_04 putative transaldolase | |
| *Specific to HDM-WSP lineage* | |
| CL6004CT6724_02 multicopper oxidase, putative | |
| CL9715CT14526_03 long-chain acyl-CoA synthetase 4 | |
| *Specific to Tibet lineage*  Null Null | |

**Table S11.** Partial Mantel test between pairwise genetic distance (*F*_ST_ / (1 - *F*_ST_)) and geographical & environmental distances conditioned with each other in Tibet lineage, HDM-WSP lineage and all populations.

|  | **Tibet Lineage** | |  | **HDM-WSP Lineage** | |  | **All populations** | |
| --- | --- | --- | --- | --- | --- | --- | --- | --- |
|  | **Mantel’s *r*** | ***P*** |  | **Mantel’s *r*** | ***P*** |  | **Mantel’s *r*** | ***P*** |
| IBD conditioned with environmental distance | **0.4896611** | **0.01** |  | **0.3393144** | **<0.001** |  | **0.6453385** | **<0.001** |
| IBE conditioned with geographical distance | -0.231604271 | 0.94 |  | **0.17993409** | **0.05** |  | **0.12726992** | **0.04** |

**Table S12.** Mantel test and partial Mantel test (conditioned with geographical distance) between pairwise genetic distance (*F*_ST_ / (1 - *F*_ST_)) and four environmental distances in Tibet lineage, HDM-WSP lineage and all populations.

|  | **Tibet Lineage** | |  | **HDM-WSP Lineage** | |  | **All populations** | |
| --- | --- | --- | --- | --- | --- | --- | --- | --- |
|  | **Mantel’s *r*** | ***P*** |  | **Mantel’s *r*** | ***P*** |  | **Mantel’s *r*** | ***P*** |
| *Mantel test* | | | | | | | | |
| bio03 | -0.008830637 | 0.5284 |  | 0.12718014 | 0.0901 |  | 0.06903403 | 0.1258 |
| bio09 | 0.04030059 | 0.3039 |  | -0.04893235 | 0.8772 |  | -0.0215991 | 0.8113 |
| prec01 | 0.04430684 | 0.3018 |  | **0.2377612** | **0.0055** |  | **0.1882733** | **0.0015** |
| prec06 | 0.1344476 | 0.1898 |  | **0.2172784** | **0.0181** |  | 0.065319805 | 0.147 |
| *partial Mantel test* | | | | | | | | |
| bio03 | -0.20333582 | 0.9391 |  | 0.07284082 | 0.1974 |  | 0.01907151 | 0.3457 |
| bio09 | 0.04818391 | 0.2761 |  | -0.03714153 | 0.816 |  | -0.02907272 | 0.8993 |
| prec01 | -0.1826748 | 0.9081 |  | **0.15783523** | **0.0409** |  | **0.2158701** | **0.0004** |
| prec06 | -0.16911801 | 0.869 |  | 0.09543843 | 0.1626 |  | 0.06457737 | 0.1544 |

**Table S13.** Correlations (Spearman’s ρ) of genetic distance (*F*_ST_ / (1 - *F*_ST_)) with geographical & environmental distances in Tibet lineage, HDM-WSP lineage and all populations, estimated by MRM (Multiple Regression on distance Matrices).

|  | **Tibet Lineage** | |  | **HDM-WSP Lineage** | |  | **All populations** | |
| --- | --- | --- | --- | --- | --- | --- | --- | --- |
|  | **ρ** | ***P*** |  | **ρ** | ***P*** |  | **ρ** | ***P*** |
| geographical distance | **0.48436995** | **0.0123** |  | **0.39511645** | **<0.001** |  | **0.655006501** | **<0.001** |
| bio03 | -0.04297302 | 0.6858 |  | 0.06878480 | 0.3006 |  | 0.001259195 | 0.9717 |
| bio09 | -0.02099007 | 0.8368 |  | 0.03128032 | 0.6007 |  | 0.044901220 | 0.1395 |
| prec01 | -0.12791566 | 0.3506 |  | **0.16786705** | **0.0222** |  | **0.135452669** | **0.0026** |
| prec06 | -0.01043626 | 0.939 |  | -0.01828982 | 0.8299 |  | -0.012257176 | 0.7337 |
| ρ2 and *P* of best model | **0.1865271** | **0.0302** |  | **0.269273** | **<0.001** |  | **0.4608219** | **<0.001** |

**Table S14.** Variable importance (%) of Generalized Dissimilarity Model correlated with geographic and environmental variables.

| **Variables** | **Tibet lineage** | **HDM-WSP lineage** | **All populations** |
| --- | --- | --- | --- |
| Geographic | 55.05 | 13.28 | 82.43 |
| bio03 | 7.81 | 0.11 | 0.11 |
| bio09 | 4.27 | 6.89 | 0.98 |
| prec01 | 0.00 | 13.42 | 5.34 |
| prec06 | 1.71 | 3.11 | 0.04 |

**Table S15.** Summary of Risk of non-Adaptedness (RONA) calculated for RCP26 and RCP85 in Tibet and HDM-WSP lineages based “Future” climatic predictions for 2050.

|  | **RCP26** | | |  | **RCP85** | | |
| --- | --- | --- | --- | --- | --- | --- | --- |
|  | **Precipitation during the dry season** | **Precipitation during the wet season** | **Longitude** |  | **Precipitation during the dry season** | **Precipitation during the wet season** | **Longitude** |
| ***Tibet lineage*** | | | | | | | |
| **LZD** | 0.0000 | 1.8948 | 0.4036 |  | 0.0000 | 2.4688 | 0.3434 |
| **GBX** | 0.1019 | 0.3218 | 0.0524 |  | 0.1019 | 0.8136 | 0.0000 |
| **MLJ** | 0.1085 | 1.3333 | 0.1884 |  | 0.1085 | 1.9418 | 0.1637 |
| **GB** | 0.0000 | 0.6836 | 0.0812 |  | 0.0000 | 1.3111 | 0.0286 |
| **MLL** | 0.1021 | 1.4936 | 0.1914 |  | 0.1021 | 2.1004 | 0.1484 |
| **SJLS** | 0.1093 | 1.5564 | 0.2053 |  | 0.1093 | 2.1288 | 0.1283 |
| **DZC** | 0.1037 | 0.4632 | 0.1126 |  | 0.1037 | 0.1158 | 0.1666 |
| **KDG** | 0.0000 | 0.7446 | 0.0536 |  | 0.0000 | 1.3713 | 0.0211 |
| **LZA** | 0.0968 | 1.4886 | 0.1499 |  | 0.0968 | 2.0965 | 0.0570 |
| **BYZ** | 0.1155 | 1.2834 | 0.0974 |  | 0.1155 | 1.9286 | 0.0143 |
| **LZ** | 0.1034 | 0.7677 | 0.0071 |  | 0.1034 | 1.4019 | 0.0690 |
| **BM** | 0.1941 | 0.2289 | 0.0899 |  | 0.1941 | 0.3375 | 0.1416 |
| **LL** | 0.1140 | 1.0570 | 0.0569 |  | 0.1140 | 1.6661 | 0.0280 |
| **MLP** | 0.1910 | 1.7012 | 0.1558 |  | 0.1910 | 2.3071 | 0.0632 |
| **BMS** | 0.0000 | 2.2482 | 0.2509 |  | 0.0000 | 2.7483 | 0.1531 |
| **BMR** | 0.1134 | 1.3508 | 0.1041 |  | 0.1134 | 1.8408 | 0.0071 |
| **BMZ**  **#SNPs** | 0.1086  54 | 0.8415  49 | 0.0351  38 |  | 0.1086  54 | 1.2985  49 | 0.0637  38 |
| **Min R^2^** | 0.0000 | 0.0000 | 0.0269 |  | 0.0000 | 0.0000 | 0.0269 |
| **Max R^2^** | 0.5093 | 0.3294 | 0.3804 |  | 0.5093 | 0.3294 | 0.3804 |
| **Average R^2^** | 0.1743 | 0.0722 | 0.2048 |  | 0.1743 | 0.0722 | 0.2048 |
| ***HDM-WSP lineage*** | | | | | | | |
| **CY** | 0.3613 | 0.1346 | 0.0103 |  | 0.7398 | 0.1346 | 0.0780 |
| **MK** | 0.4762 | 0.0618 | 0.0097 |  | 0.9276 | 0.0618 | 0.0589 |
| **DQ** | 0.0747 | 0.0000 | 0.0770 |  | 0.6754 | 0.0665 | 0.0196 |
| **DR** | 0.6902 | 0.0337 | 0.0201 |  | 1.3076 | 0.0337 | 0.0360 |
| **ZD** | 0.8313 | 0.1964 | 0.1816 |  | 1.4650 | 0.1291 | 0.1223 |
| **XG** | 0.5671 | 0.1503 | 0.1927 |  | 1.1840 | 0.1278 | 0.1205 |
| **XC** | 1.1724 | 0.0332 | 0.0403 |  | 0.9469 | 0.0332 | 0.0051 |
| **LJ** | 0.9738 | 0.3012 | 0.3804 |  | 1.1405 | 0.2685 | 0.2690 |
| **DC** | 0.6690 | 0.0000 | 0.0360 |  | 0.4704 | 0.0000 | 0.0249 |
| **YJ** | 1.1753 | 0.0322 | 0.0920 |  | 0.4993 | 0.0322 | 0.0748 |
| **RT** | 0.1712 | 0.0337 | 0.0398 |  | 0.0144 | 0.0576 | 0.0847 |
| **DF** | 0.9794 | 0.0320 | 0.0888 |  | 0.7557 | 0.0320 | 0.1038 |
| **KD** | 0.5151 | 0.0333 | 0.0811 |  | 0.3651 | 0.0333 | 0.0708 |
| **YYP** | 1.2384 | 0.0673 | 0.2081 |  | 1.3512 | 0.0000 | 0.1874 |
| **JC** | 1.3258 | 0.0000 | 0.0561 |  | 1.5139 | 0.0000 | 0.1008 |
| **MEK** | 0.4385 | 0.0000 | 0.1009 |  | 0.6577 | 0.0000 | 0.1574 |
| **XJ** | 1.5855 | 0.1310 | 0.1388 |  | 1.4351 | 0.1635 | 0.1837 |
| **LX** | 1.0607 | 0.0000 | 0.0862 |  | 1.2484 | 0.0000 | 0.1259 |
| **HS** | 0.7967 | 0.0337 | 0.0736 |  | 0.9354 | 0.0337 | 0.1313 |
| **MX** | 0.7810 | 0.0669 | 0.0660 |  | 0.8879 | 0.0669 | 0.1202 |
| **WC** | 0.4159 | 0.0337 | 0.0738 |  | 0.5340 | 0.0337 | 0.1154 |
| **PW**  **#SNPs** | 2.4023  36 | 0.0000  31 | 0.0103  25 |  | 2.5151  36 | 0.0000  31 | 0.0514  25 |
| **Min R^2^**  **Max R^2^** | 0.0000 | 0.0000 | 0.0045 |  | 0.0000 | 0.0000 | 0.0045 |
|  | 0.4627 | 0.4331 | 0.4165 |  | 0.4627 | 0.4331 | 0.4165 |
| **Average R^2^** | 0.1119 | 0.0434 | 0.1211 |  | 0.1119 | 0.0434 | 0.1211 |

**Table S16.** Summary of Risk of non-Adaptedness (RONA) calculated for RCP26 and RCP85 in Tibet and HDM-WSP lineages based “Future” climatic predictions for 2070.

|  | **RCP26** | | |  | **RCP85** | | |
| --- | --- | --- | --- | --- | --- | --- | --- |
|  | **Precipitation during the dry season** | **Precipitation during the wet season** | **Longitude** |  | **Precipitation during the dry season** | **Precipitation during the wet season** | **Longitude** |
| ***Tibet lineage*** | | | | | | | |
| **LZD** | 0.0000 | 1.9307 | 0.5375 |  | 0.0000 | 2.7557 | 0.2572 |
| **GBX** | 0.1019 | 0.3383 | 0.1236 |  | 0.1019 | 1.1080 | 0.0750 |
| **MLJ** | 0.1085 | 1.3691 | 0.2754 |  | 0.1085 | 2.2640 | 0.1262 |
| **GB** | 0.0000 | 0.9257 | 0.1513 |  | 0.0000 | 1.5965 | 0.0588 |
| **MLL** | 0.1021 | 1.7433 | 0.2688 |  | 0.1021 | 2.6386 | 0.0473 |
| **SJLS** | 0.1093 | 1.7710 | 0.2846 |  | 0.1093 | 2.7016 | 0.0214 |
| **DZC** | 0.1037 | 0.4327 | 0.0799 |  | 0.1037 | 0.3291 | 0.2111 |
| **KDG** | 0.0000 | 0.7446 | 0.1137 |  | 0.0000 | 1.6570 | 0.0873 |
| **LZA** | 0.0968 | 1.4886 | 0.2303 |  | 0.0968 | 2.3835 | 0.0375 |
| **BYZ** | 0.1155 | 1.3191 | 0.1639 |  | 0.1155 | 2.1797 | 0.0840 |
| **LZ** | 0.1034 | 0.7677 | 0.0603 |  | 0.1034 | 1.6500 | 0.1284 |
| **BM** | 0.1941 | 0.2289 | 0.0500 |  | 0.1941 | 0.5187 | 0.1668 |
| **LL** | 0.1140 | 1.2720 | 0.1067 |  | 0.1140 | 1.9531 | 0.1115 |
| **MLP** | 0.1910 | 1.9136 | 0.2181 |  | 0.1910 | 2.8436 | 0.0345 |
| **BMS** | 0.0000 | 2.3196 | 0.2973 |  | 0.1082 | 3.2855 | 0.0603 |
| **BMR** | 0.1134 | 1.4206 | 0.1382 |  | 0.0000 | 2.3745 | 0.0494 |
| **BMZ**  **#SNPs** | 0.1086  54 | 0.9085  49 | 0.0673  38 |  | 0.0000  54 | 1.7628  49 | 0.0923  38 |
| **Min R^2^** | 0.0000 | 0.0000 | 0.0269 |  | 0.0000 | 0.0000 | 0.0269 |
| **Max R^2^** | 0.5093 | 0.3294 | 0.3804 |  | 0.5093 | 0.3294 | 0.3804 |
| **Average R^2^** | 0.1743 | 0.0722 | 0.2048 |  | 0.1743 | 0.0722 | 0.2048 |
| ***HDM-WSP lineage*** | | | | | | | |
| **CY** | 0.4480 | 0.1346 | 0.0051 |  | 1.2321 | 0.1010 | 0.0723 |
| **MK** | 0.5514 | 0.0618 | 0.0103 |  | 1.4540 | 0.0332 | 0.0349 |
| **DQ** | 0.1438 | 0.0337 | 0.0770 |  | 1.0588 | 0.0989 | 0.0397 |
| **DR** | 0.7545 | 0.0337 | 0.0393 |  | 1.7563 | 0.0000 | 0.0103 |
| **ZD** | 0.8313 | 0.1627 | 0.1784 |  | 1.8787 | 0.1291 | 0.1457 |
| **XG** | 0.5999 | 0.1278 | 0.1927 |  | 1.2937 | 0.1007 | 0.1443 |
| **XC** | 1.0972 | 0.0332 | 0.0738 |  | 0.3899 | 0.0332 | 0.0290 |
| **LJ** | 0.9738 | 0.2685 | 0.3560 |  | 1.5978 | 0.2353 | 0.3032 |
| **DC** | 0.6349 | 0.0000 | 0.0562 |  | 0.0989 | 0.0000 | 0.0103 |
| **YJ** | 1.0625 | 0.0322 | 0.1806 |  | 0.3499 | 0.0634 | 0.0961 |
| **RT** | 0.1269 | 0.0337 | 0.1056 |  | 0.5733 | 0.0576 | 0.0653 |
| **DF** | 0.8666 | 0.0320 | 0.1765 |  | 0.2461 | 0.0587 | 0.0988 |
| **KD** | 0.4776 | 0.0333 | 0.1901 |  | 0.1222 | 0.0435 | 0.0862 |
| **YYP** | 1.2384 | 0.0336 | 0.2844 |  | 1.8778 | 0.0000 | 0.1998 |
| **JC** | 1.3634 | 0.0000 | 0.1325 |  | 2.1157 | 0.0147 | 0.0660 |
| **MEK** | 0.5115 | 0.0000 | 0.1746 |  | 1.2219 | 0.0260 | 0.1087 |
| **XJ** | 1.5479 | 0.1310 | 0.2599 |  | 0.8710 | 0.1965 | 0.1423 |
| **LX** | 1.0607 | 0.0000 | 0.1491 |  | 1.8118 | 0.0337 | 0.0812 |
| **HS** | 0.7632 | 0.0337 | 0.1503 |  | 1.4906 | 0.0000 | 0.0633 |
| **MX** | 0.7462 | 0.0669 | 0.1318 |  | 1.4713 | 0.0998 | 0.0510 |
| **WC** | 0.3864 | 0.0000 | 0.1432 |  | 1.0419 | 0.0000 | 0.0697 |
| **PW**  **#SNPs**  **Min R^2^** | 2.2520  36  0.0000 | 0.0337  31  0.0000 | 0.0669  25  0.0045 |  | 3.0417  36  0.0000 | 0.0337  31  0.0000 | 0.0360  25  0.0045 |
| **Max R^2^** | 0.4627 | 0.4331 | 0.4165 |  | 0.4627 | 0.4331 | 0.4165 |
| **Average R^2^** | 0.1119 | 0.0434 | 0.1211 |  | 0.1119 | 0.0434 | 0.1211 |
